# Endoglin regulates mural cell adhesion in the circulatory system

**DOI:** 10.1007/s00018-015-2099-4

**Published:** 2015-12-08

**Authors:** Elisa Rossi, David M. Smadja, Elisa Boscolo, Carmen Langa, Miguel A. Arevalo, Miguel Pericacho, Luis Gamella-Pozuelo, Alexandre Kauskot, Luisa M. Botella, Pascale Gaussem, Joyce Bischoff, José M. Lopez-Novoa, Carmelo Bernabeu

**Affiliations:** 1 Centro de Investigaciones Biológicas, Consejo Superior de Investigaciones Científicas (CSIC), c/Ramiro de Maeztu 9, 28040 Madrid, Spain; 2 Centro de Investigación Biomédica en Red de Enfermedades Raras (CIBERER), 28040 Madrid, Spain; 3 Paris Descartes University, Sorbonne Paris Cite, Paris, France; 4 Hematology Department, AP-HP, Hôpital Européen Georges Pompidou, Paris, France; 5 Faculté de Pharmacie, Inserm UMR-S1140, Paris, France; 6 Department of Surgery, Harvard Medical School, Children’s Hospital, Boston, MA 02115 USA; 7 Departamento de Anatomía e Histología Humanas, Facultad de Medicina, Universidad de Salamanca, 37007 Salamanca, Spain; 8 Instituto de Investigaciones Biomédicas de Salamanca (IBSAL), 37007 Salamanca, Spain; 9 Departamento de Fisiología y Farmacología, Unidad de Fisiopatología Renal y Cardiovascular, Universidad de Salamanca, 37007 Salamanca, Spain; 10 Inserm UMR-S1176, Le Kremlin Bicêtre, Paris, France; 11 Université Paris Sud, Le Kremlin Bicêtre, Paris, France

**Keywords:** Blood vessels, Tubulogenesis, Cell adhesion, TGF-β, HHT, Kidney

## Abstract

The circulatory system is walled off by different cell types, including vascular mural cells and podocytes. The interaction and interplay between endothelial cells (ECs) and mural cells, such as vascular smooth muscle cells or pericytes, play a pivotal role in vascular biology. Endoglin is an RGD-containing counter-receptor for β1 integrins and is highly expressed by ECs during angiogenesis. We find that the adhesion between vascular ECs and mural cells is enhanced by integrin activators and inhibited upon suppression of membrane endoglin or β1-integrin, as well as by addition of soluble endoglin (SolEng), anti-integrin α5β1 antibody or an RGD peptide. Analysis of different endoglin mutants, allowed the mapping of the endoglin RGD motif as involved in the adhesion process. In *Eng* ^+/−^ mice, a model for hereditary hemorrhagic telangectasia type 1, endoglin haploinsufficiency induces a pericyte-dependent increase in vascular permeability. Also, transgenic mice overexpressing SolEng, an animal model for preeclampsia, show podocyturia, suggesting that SolEng is responsible for podocytes detachment from glomerular capillaries. These results suggest a critical role for endoglin in integrin-mediated adhesion of mural cells and provide a better understanding on the mechanisms of vessel maturation in normal physiology as well as in pathologies such as preeclampsia or hereditary hemorrhagic telangiectasia.

**Electronic supplementary material:**

The online version of this article (doi:10.1007/s00018-015-2099-4) contains supplementary material, which is available to authorized users.

## Introduction

The circulatory system is walled off by different cell types, including vascular mural cells and podocytes. Blood vessels are composed of two cell types: endothelial cells (ECs) and mural cells which are commonly subdivided into vascular smooth muscle cells (VSMCs) and pericytes [1]. The recruitment of pericytes and VSMCs along the endothelial tube networks represents a critical event controlling capillary remodeling, maturation and stabilization [2, 3]. Overall, vascular mural cells have demonstrated to be important for angiogenesis, structural integrity of the microvasculature and blood flow regulation. Several cytokines are involved in the regulation of mural cell function [4]. Secretion by ECs of platelet-derived growth factor (PDGF) regulates pericyte recruitment to vessels and vascular pattern formation [5, 6]. Also, CXCL12, upregulated by hypoxia [7], promotes recruitment, vascular remodeling and differentiation of pericytes [8, 9]. Transforming growth factor-β (TGF-β) is expressed by ECs and pericytes during angiogenesis, and its signaling pathway is essential for pericyte differentiation and recruitment to nascent vessels, as well as for vessel maturation [10]. Mural and endothelial cells express several members of the integrin adhesion family of proteins, which are involved in cell–cell and cell–matrix adhesion processes required for angiogenesis, vascular stability and vessel maturation [11, 12]. Moreover, β1 integrin family members, including the fibronectin receptor α5β1, can be activated by proangiogenic chemokines such as CXCL12 and are major determinants of the mural cell phenotype, controlling cell adhesion, spreading, and blood vessel wall stability [13–16]. Podocytes (or glomerular visceral epithelial cells) are another type of mural cells, located in the glomerular tuft of the kidney, that wrap around the capillaries of the glomerulus, contribute to capillary stabilization and retain large proteins in the blood by impeding its filtration through the wall of glomerular capillaries to the Bowman’s space. Podocytes are exposed to considerable mechanical stress produced by the plasma filtration [17] and to maintain a functional filtration barrier, they must adhere strongly to the glomerular basement membrane (GBM) [18, 19]. Podocyte adhesion is mediated, at least, by the extracellular engagement of integrin α3β1 to the GBM component laminin-52117. In spite of the emerging relevant role of integrins, as cell adhesion receptors in vascular biology [11, 13, 20], their involvement in the interaction between ECs and mural cells remains largely unexplored.

Endoglin (Eng) is an endothelial membrane receptor that, in addition to act as an auxiliary partner protein in the TGF-β receptor complex, can function as a ligand for leukocyte integrins [16, 21]. Structurally, the extracellular region of endoglin contains two distinct domains: (1) a zona pellucida (ZP) juxtamembrane domain that in human endoglin displays the prototypic arginine-glycine-aspartic acid (RGD) motif involved in integrin-based interactions [16, 22, 23]; and (2) the NH2-terminal orphan domain involved in binding to BMP9, a member of the TGF-β superfamily [23–25]. Endoglin is highly expressed by ECs during neoangiogenic processes at the same time as mural cells recruitment to vessels occurs, and plays a critical physiological role in the cardiovascular system [21]. Mutations in the endoglin gene are responsible for a vascular disorder known as Hereditary Hemorrhagic Telangectasia type 1 (HHT1) characterized by recurrent epistaxis, telangiectasia and arteriovenous malformations [26, 27]. Several lines of experimental evidence support the involvement of endoglin in the mural cell recruitment to ECs: (1) mice lacking endoglin die by gestational day 11.5 from defective vascular development [28–30]; (2) loss of endoglin in these *Eng* ^−/−^ mice causes poor vascular smooth muscle development and arrested endothelial remodeling [28, 31], suggesting that VSMCs play a role in regulating endothelial organization and is involved in the pathogenesis of HHT1; and (3) thalidomide is used to treat the epistaxis of HHT patients through a mechanism involving the activation of mural cells, leading to the embracing and stabilization of small blood vessels [32]. Moreover, endoglin cleavage produced by MMP14 at a juxtamembrane site gives rise to the soluble form of endoglin (SolEng) whose levels are significantly increased in preeclampsia [33], a systemic syndrome of pregnancy clinically characterized by hypertension, proteinuria and high levels of SolEng in plasma and urine [34, 35]. Podocyturia has been reported in patients with preeclampsia, although the underlying mechanism of this observation remains to be elucidated [36, 37].

Pericytes, VSMCs and podocytes have in common that they wall off the circulatory system via integrins. Based on the physical and functional association between endoglin and β1 integrins [16, 38, 39], our working hypothesis is that endoglin may act as an adhesion molecule involved in the integrin-mediated binding of vascular mural cells and podocytes. Here, we find that membrane and SolEng regulate these adhesion processes by interacting with β1-integrins, at least, via its RGD motif. These results provide a better understanding on the mechanisms of vessel development and maturation in vascular pathophysiology, including preeclampsia and HHT1.

## Materials and methods

### Cell lines, primary cultures, antibodies and other reagents

The Jurkat (T cell lymphoblast-like) cell line was cultured in RPMI 1640 supplemented with 10 % heat inactivated fetal calf serum (FCS). Human umbilical vein endothelial cells (HUVECs), and human aortic endothelial cells (HAECs) (Lonza, Walkersville, USA) were grown on gelatin (Sigma) coated wells using EBM-2 medium, supplemented with EGM-2 SingleQuots (Lonza) and 10 % FCS. Umbilical artery smooth muscle cells (UASMCs; Lonza) were grown in SmGM2 bullet kit medium (Lonza), supplemented with 10 % FCS. Endothelial colony-forming cells (ECFCs) exhibit features of a true endothelial progenitor population [40, 41]. ECFCs were isolated as described [40–42], and grown on gelatin coated wells using EBM-2 medium, supplemented by 20 % FCS and by EGM-2 SingleQuots (Lonza). Bone marrow-derived mesenchymal progenitor cells (bmMPCs) [43, 44] were grown on fibronectin (Millipore) coated wells using EBM-2 medium, supplemented by 20 % FCS, rhFGF-B, R3-IGF-1, ascorbic acid, and GA-1000 (Lonza). bmMPCs were differentiated into pericytes by seeding bmMPCs together with ECFCs at a ratio 1:1 at a total density of 10^4^ cells/cm^2^ on fibronectin-coated plates in EBM-2 medium containing 20 % FCS. All cell types were cultured in a 5 % CO_2_ atmosphere at 37 °C. Antibodies, RGD-related peptides and recombinant proteins are described in Online Resource Supplemental Material.

### Expression and silencing vectors and cell transfections

The expression vector encoding HA-tagged full length endoglin and the derived truncated construct 437/586-Endo in pDisplay (Invitrogen) have been previously reported [45]. The pCEXV-EndoL plasmid, encoding human L-endoglin [46], was used to derive by PCR amplification a truncated endoglin construct containing the zona pellucida domain (ZPD; amino acids 340–658). The resulting ZPD-Endo-pDisplay vector was used to generate, by site directed mutagenesis a mutant of the RGD motif, where the Asp401 residue is replaced by Ala, leading to the ZPD-Endo-RGA-pDisplay vector. All endoglin constructs expressed from pDisplay contain the influenza hemagglutinin epitope HA at the NH_2_ terminus. To silence endoglin expression, siRNAs (s4677 and s4679) and scrambled siRNAs (AM4611 and AM4613), as negative controls (Ambion, Life Technology), were used. Nucleofections with expression and silencing vectors were performed following the manufacturer’s instructions with Amaxa nucleofector kits VCA-1003 (Jurkat cell line), VPB-1002 (HUVECs), and VPI-1004 (UASMCs) from Lonza using the Nucleofector I (AMAXA, Germany). Silencing experiments were carried out by simultaneously nucleofecting two endoglin siRNAs (s4677 and s4679) using scrambled siRNAs #1 (AM4611) and #2 (AM4613), as negative controls. Alternatively, silencing of endoglin was performed using endoglin siRNA (sc-35302; Santa Cruz Biotech) and scrambled siRNA (AllStars; Qiagen #1027280), as a negative control, in the presence of PrimeFect siRNA transfection reagent and PrimeFect diluent (PA3269 and PA3271; Lonza). Silencing of β1 integrin was performed using β1 integrin siRNA (sc-35674; Santa Cruz Biotech) and scrambled siRNA (AllStars; Qiagen #1027280), as a negative control, in the presence of PrimeFect siRNA transfection reagent and PrimeFect diluent (PA3269 and PA3271; Lonza). To monitor the suppression efficiency, immunofluorescence flow cytometry to detect endoglin or β1 integrin expression was performed.

### Binding assays of SolEng to cells

The chimeric construct EndoEC-Fc, encoding the extracellular domain of endoglin fused to the Fc fragment of IgG has been described [47]. Exponentially growing UASMCs were incubated either in the presence or absence of CXCL12 (200 ng/mL) or MnCl_2_ (200 μM) for 20 min at 37 °C. Cells were washed with PBS and incubated with different combinations of EndoEC-Fc, RGD peptide (1 mM) or DGR peptide (1 mM) in 2 % AB^+^ human serum for 10 min at 37 °C. Samples were then incubated with FITC-labeled secondary antibody anti-human IgG for 1 h at 4 °C, washed with PBS and fixed with 10 % formaldehyde. The Vectashield mounting medium for fluorescence with DAPI (H-1200, Vector) was used to counterstain the samples and analyze them by confocal microscopy (Confocal Laser Scanning Microscope, CLSM, Leica TCS SP5) and video recording (Leica Las AF Lite).

### Immunofluorescence flow cytometry

Cells in suspension were incubated for 30 min at 4 °C with 2 % human AB^+^ serum and then for 1 h at 4 °C with the primary antibody. After two washes with cold PBS, samples were incubated with the secondary antibody (Alexa 488 anti-mouse). Finally, cells were washed twice, and their mean fluorescence intensity (MFI) was measured in a Coulter Epics XL flow cytometer (Beckman Coulter). When necessary, cells nucleofected with green fluorescent protein (GFP) were also analyzed by fluorescence flow cytometry. For double fluorescence analysis, cells were selected on basis of forward and side scatter characteristics and the results were represented in a dot plot showing events (cells) for the two fluorescence signals (675 nm for Alexa 647 and 525 nm for GFP), using an FC-500 flow cytometer (Beckman Coulter).

### Immunofluorescence of 2D co-culture

Culture chamber slides were coated with fibronectin and seeded with ECFC and bmMPC at a 1:1 ratio. After in vitro co-culture for 7 days, bmMPC differentiate into VSMC/pericytes [43]. Once differentiated, cells were fixed with cold pure methanol on ice for 10 min. For immunostaining of ECFC, samples were incubated with a mAb anti-human von Willebrand factor (Dako) for 1 h at room temperature, followed by incubation with the secondary antibody Texas Red anti-mouse IgG (Vector) for 1 h at room temperature. Differentiated bmMPCs were incubated with anti-human calponin (Abcam), anti-human Sm22α (Abcam), anti-PDGFRb (Santa Cruz), anti-human NG2 (R&D Systems), anti-human αSMA (Sigma) or a negative control antibody (Santa Cruz). After washing samples were incubated with the appropriate FITC-labeled secondary antibody, and mounted using Vectashield with DAPI (Vector).

### Cell adhesion assays

Cultures of UASMCs in 24-well plates were used as a cell substrate in adhesion assays with the Jurkat cell line. When UASMCs were 90 % confluent, Jurkat cells nucleofected with human endoglin and GFP constructs were added to the wells. After 1-h incubation, wells were rinsed with PBS and photographed by confocal microscopy. Then, bound cells were lysed and their fluorescence was measured. When necessary, UASMCs were stained with a red vital stain (CellTracker™ Red CMTPX, Life Technologies) for 30 min at 37 °C, and then washed with PBS prior to the cell adhesion assay. The quantification of substrate adherence capacity was carried out by a fluorescent analyser (Varioskan Thermo-Fisher Scientific), selecting the appropriate wavelength (GFP 494–515 nm). A similar method was used to quantify the adhesion of pericytes to endothelial cells. Briefly, HUVEC monolayers or pericyte monolayers stained with CMTPX were incubated with pericytes or HUVECs, respectively, previously labeled with the green stain CSFE (CellTracker™, Life Technologies) in the presence or in the absence of SolEng (50 ng/mL), CXCL12 (100 ng/mL), RGD, RGDK, or DGR peptides (1 mM), or MnCl_2_ (200 ng/mL). After 1 h of incubation, wells were subjected to lysis and adhesion of pericytes to HUVECs was measured using the Varioskan equipment selecting the appropriate wavelength (CSFE: 494–515 nm). Images of cell–cell adhesion were obtained by confocal microscopy (Confocal Laser Scanning Microscope, CLSM, Leica TCS SP5). Quantification of bound cells was carried out by measuring the surface area of the cells using ImageJ Version 1.46 software. When required, UASMCs or HAECs were preincubated with 100 ng/mL CXCL12 for 30 min at 37 °C, prior to CSFE labeling and cell adhesion assays. The concentrations of cycloheximide and monensin were selected from an initial dose-dependent study to exclude possible toxic effects of the drugs.

### Phosphorylation and western blot studies

Individual and combined cultures of UASMCs and HAECs were incubated in the absence or presence of 1 μg/mL SolEng. At different time points, adherent cells were lysed in RIPA buffer, and proteins were subjected to SDS-PAGE and transferred to nitrocellulose. The membranes were incubated with the primary antibodies anti-p-FAK (Tyr925; BD Biosciences), anti-p-Akt (Ser473; Cell Signalling) and anti-actin (Sigma). Immunoreactive bands were visualized using enhanced chemiluminescence detection reagents (Pierce). Images of chemiluminescent signals were captured using G:BOX Chemi XT16 Image Systems and quantified using Gene Tools version 4.0.0.0 (Syngene). The presence of HA-tagged SolEng in concentrated urine samples was detected by Western blot analysis with anti-endoglin (P4A4) and anti-HA antibodies. Endoglin protein bands were visualized with a ChemiDoc™ XRS+ equipment (Bio-Rad) and their intensity was quantified using Image Lab™ software.

### 3D coculture of vascular ECs and mural cells

3D cocultures of endothelial cells and UASMCs at a 4:1 ratio, respectively, were performed using Matrigel (BD Bioscience). When necessary, ECs stained in red (CMTPX) and UASMCs stained in green (CSFE) were co-cultured. Similarly, HUVECs, HAECs, or UASMCs nucleofected with siRNA/GFP were resuspended and cocultured in Matrigel. Endothelial cells (HUVECs/HAECs/ECFCs) and mural cells (UASMC, bmMPC) were resuspended in a mixture of Matrigel:Medium (1:1) in a total volume of 100 μL/well. For in vitro assays, 4-well glass slides with a 1.7 cm^2^ growing area per well (Millipore) were used. Additives were added at time 0 and cocultures were monitored for 6 h at 37 °C. After 4 h of incubation, photographs of the slides were taken. For studying the potential role of integrins we used the anti-β1 mAbs TS2/16 and LIA1/2. As a global integrin activator MnCl_2_ at 200 μM was used. The macrocyclic compound AMD3100 octahydrochloride (Sigma-Aldrich), inhibitor of the CXCR4 alpha-chemokine receptor (5 μM), human SolEng (1 μg/mL), RGD (Arg-Gly-Asp; 1 mM), PDGF-BB (10 ng/mL) and CXCL12 (200 ng/mL) were also used.

### Immunohistochemistry and ELISA

Sections (3 μm) from kidneys, lungs and Matrigel plugs were cut and stained with hematoxylin and eosin or with Masson’s trichrome stain. For immunohistochemical analysis, sections were deparaffinated in xylene and rehydrated in a graded series of ethanol. The antigen-retrieval process was carried out by a 3-min microwave incubation of sections with citrate solution (BioGenex). Endogenous peroxidase was blocked by incubation in 3 % hydrogen peroxide and sections were incubated with anti-αSMA (Leica Biosystems), anti-podocin (Abcam), anti-Willms Tumor (WT1) (sc-192; Santa Cruz Biotechnology), anti-CD31 (Leica Biosystems), mouse monoclonal anti-CD105 (Dako) or anti-CD68 (Dako). Then, sections were washed in PBS and incubated with the Novolink Polymer Detection System (Novocastra), followed by reaction with 3,3′diaminobenzidine as chromogen. Negative controls were performed in the absence of the primary antibody. For ThinPrep cytology, urine cytological slides were prepared by using a liquid-based method technique following the manufacturer’s guidelines (ThinPrep 2000; Cytyc) and stained using either the standard hematoxylin technique or immunohistochemistry against αSMA as above. Urine samples were used to determine the concentration of SolEng (R&D Systems), nephrin (Exocell) and podocalyxin (Wuhan EIAAB Science), using ELISA kits.

### Mice and in vivo assays

For Matrigel plug assays, ECFCs and bmMPCs (1:1 ratio; total cells 3 × 10^6^) were resuspended in 200 μL of Matrigel (BD Bioscience) either in the absence or in the presence of human SolEng and subcutaneously injected into 6-week-old male nude mice (*n* = 13). Parallel assays were carried out in the absence of cells. After 7 days, intact Matrigel plugs were carefully exposed, fixed, and embedded in paraffin for histological examination. Three different sections of each plug were analyzed and blood vessels were quantified by hematoxylin and eosin staining. Transgenic C57BL/6J mice (12–20 weeks of age) with recurrent backcrossing, expressing an HA-tagged soluble form of endoglin, driven by a ubiquitous actin promoter (*Sol.Eng* ^+^) [35], were used for immunohistochemistry of the kidney and lung. Representative tissue stainings of *Sol.Eng* ^+^ (*n* = 17) and WT (*n* = 17) mice are shown in the figures. Urine from *Sol.Eng* ^+^ (*n* = 10) and WT (*n* = 10) mice was collected in metabolic cages or obtained directly from the bladder. Urinary cytology was performed using the ThinPrep technique. The concentration of endoglin and the podocyte markers nephrin and podocalyxin was measured by ELISA and urinary cells were stained with podocyte-specific antibodies. Vascular permeability studies were carried out in *Eng* ^+*/*−^ mice and their *Eng* ^+/+^ littermates [29, 48]. Mice were anesthetized with 1.2 % isoflurane and perfused through the jugular vein with a solution of fluorescein isothiocyanate-dextran (FITC-dextran; 2 × 10^6^ kDa; Sigma-Aldrich) at 100 mg/kg body weight. After 2 h, mice were killed by cervical dislocation and eyes were removed and fixed in 4 % paraformaldehyde for 1 h. Eyes were dissected and the neuroretinas (*n* = 19) were isolated and mounted with antifade solution (ProLong gold, Life Technologies). Retinas were photographed using a fluorescence microscope (Axiovert 200 M, Zeiss). All procedures were approved by the Animal Care and Use Committees of the University of Salamanca and University of Paris. Also, mice were cared for in accordance with the standards established in the National Institutes of Health Guide for the Care and Use of Laboratory Animals.

### Cell quantification and statistical analysis

Binding of UASMCs to HUVECs in angiogenesis assays was quantified by measuring the intensity profile using fluorescence confocal microscopy (SP5, Leica). Fluorescence images were transformed in binary system by ImageJ to quantify the percentage of cell adhesion. In adhesion assays, bound fluorescently labeled cells were lysed and quantification was carried out using a Varioskan plate reader. All values are expressed as mean ± standard deviation. Results are representative of, at least, five independent experiments in triplicate samples. Statistical analysis was performed using a one-way ANOVA and Bonferroni post-test. A value of *p* < 0.05 (*) was considered statistically significant and a value of *p* < 0.005 (**) or *p* < 0.001 (***) was considered statistically highly significant.

An expanded “Materials and methods” section is included in the Online Resource Supplemental Material.

## Results

### Endoglin is involved in the interaction between endothelial and mural cells

**Fig. 1:**
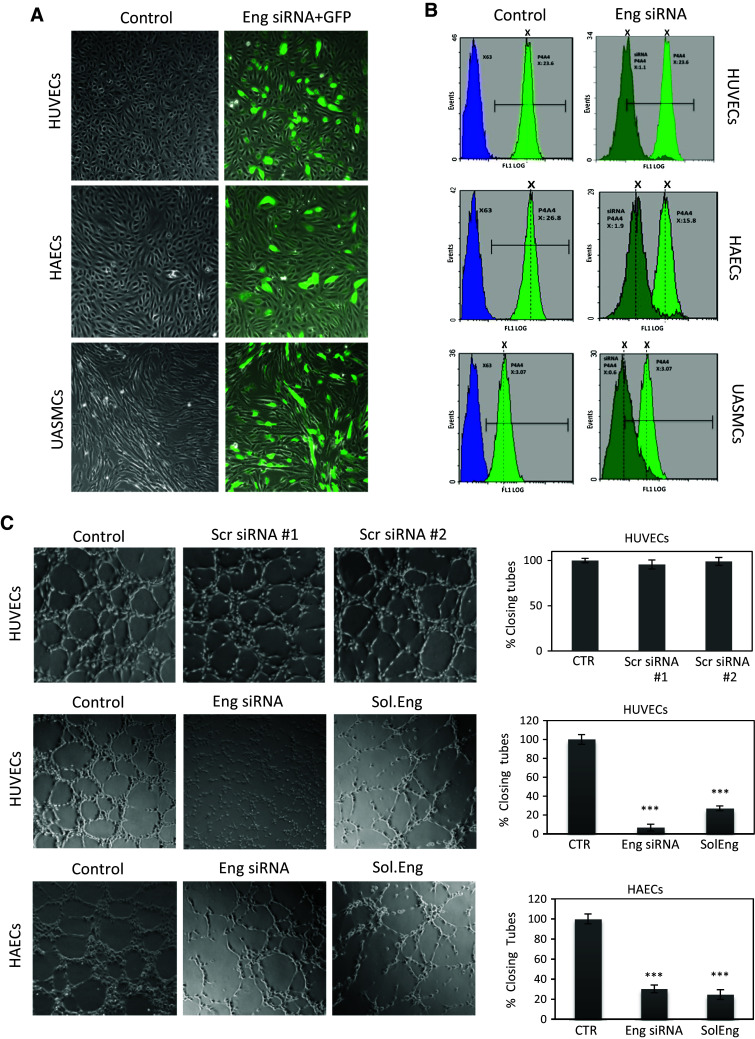
Endoglin silencing in endothelial and smooth muscle cells. **a** Primary cultures of HUVECs, HAECs and UASMCs (p4–8) were untreated (control), nucleofected with endoglin (Eng) specific siRNA (#s4677 and #s4679) and GFP (*green stain*), or nucleofected with scrambled siRNA (#AM4611 and #AM4613) and GFP (siRNA control). After 48 h, cells were observed by confocal microscopy. Cells cotransfected with GFP are visualized by their *green* fluorescence. **b** Flow cytometry analysis. Primary cultures of HUVECs, HAECs and UASMCs were untreated (control) or nucleofected with Eng siRNA or scrambled siRNA #AM4611 and #AM4613 (siRNA control). After 48 h, cells were analyzed by immunofluorescence flow cytometry with anti-endoglin mAb P4A4 (*green* histograms) or a negative control mAb (X63; *blue histograms*). The mean fluorescence intensity (MFI) of each sample is indicated. Endoglin expression is decreased upon transfection with specific siRNA in all cells. Cells nucleofected with scrambled siRNA showed the same endoglin expression levels as untreated cells (data not shown). **c** HUVECs and HAECs were incubated in matrigel to analyze tube formation. Confocal microscopy of untreated cells (control), nucleofected for 48 h with scrambled siRNA (AM4611, #1; AM4613, #2) or Eng siRNA (#s4677 and #s4679), or incubated with soluble endoglin (Sol.Eng) are shown. The histogram on the *right* indicates the percentage, respect to the control sample (100 %), of closing tubes under each experimental condition. Samples were in triplicates and the mean of the control condition was given the arbitrary value of 100. The average of five different experiments is shown. The statistical significance respect to control value (CTR) is indicated (***p < 0.001)

**Fig. 2:**
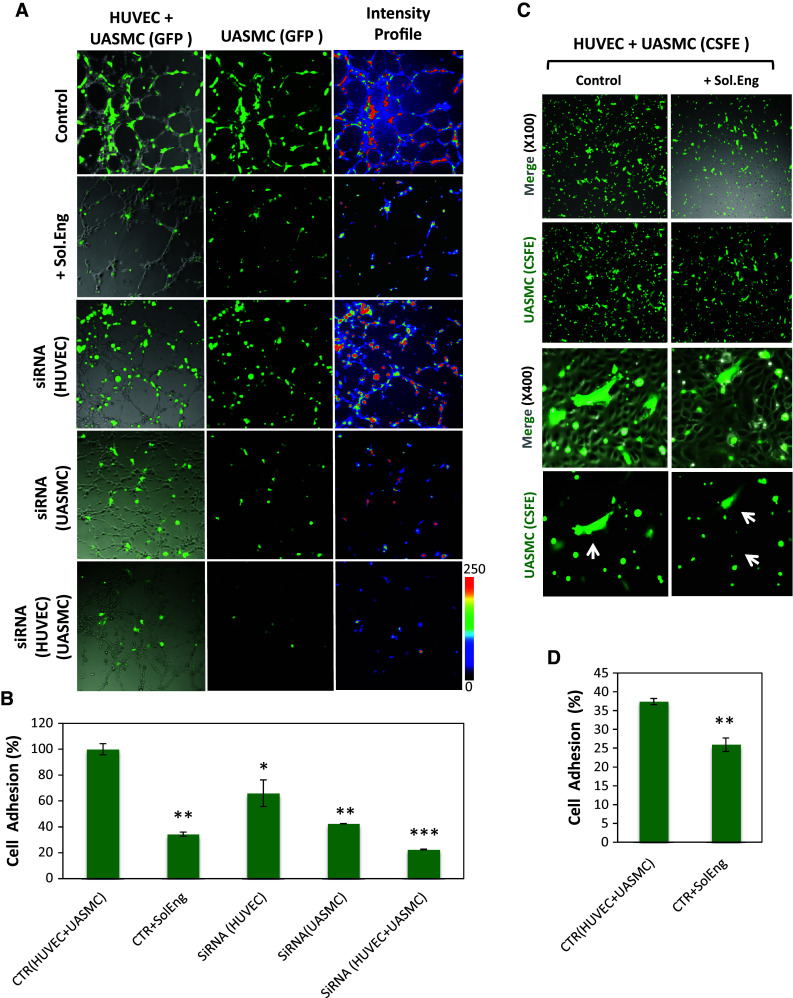
Role of endoglin in adhesion between VSMCs and ECs. **a**, **b** Cell–cell adhesion in angiogenesis assays. **a** UASMCs were transfected with GFP and cocultured with unlabeled HUVECs at a 1:4 ratio in matrigel to analyze mural cell adhesion to ECs. Confocal microscopy of untreated cells (control) and cells incubated with soluble endoglin (Sol.Eng) or nucleofected with Eng siRNA are shown in representative photographs. The intensity of the staining according to the *color scale* (0–250) indicates mural cell adhesion to endothelial cells in 3D co-culture **b** Quantification of UASMCs binding to ECs was carried out by measuring the intensity profile using fluorescence confocal microscopy (SP5, Leica). The mean area in percentage, representing mural cell adhesion measured in different fields, is indicated. Samples were in triplicates and the mean of the control condition was given the arbitrary value of 100. The average of five different experiments is shown. **c**, **d** Cell adhesion assay. **c** HUVEC monolayers were incubated with UASMCs previously labeled with CSFE in the absence or in the presence of soluble endoglin. After 1 h incubation, wells were washed and the cells were visualized by confocal microscopy. **d** Binding of UASMCs to HUVECs in **c** was quantified by measuring the intensity profile using fluorescence confocal microscopy (SP5, Leica). The average of four independent experiments is shown. The statistical significance respect to control value (CTR) is indicated. *p < 0.05; **p < 0.005; ***p < 0.001

We have previously demonstrated that endothelial endoglin is involved in integrin-mediated cell adhesion [16]. To assess whether endoglin is involved in adhesion between endothelial cells and mural cells, endoglin suppression experiments were carried out (Fig. 1). Transfection of cells with siRNA for endoglin was monitored by using a GFP expression vector (Fig. 1a) as well as by immunofluorescence flow cytometry using antibodies to endoglin (Fig. 1b). Endoglin was found to be expressed at the surface of cultured endothelial cells (HAEC, human aortic endothelial cells; and HUVEC, human umbilical vein endothelial cell) and umbilical artery smooth muscle cells (UASMCs) (Fig. 1b, left panels). Noteworthy, basal endoglin expression levels were much higher in cultured HUVECs or HAECs than in UASMCs or bmMPCs (Fig. 1b; Online Resource Supplemental Fig. 1B, C). As previously reported for HUVECs [49], wound healing assays of HAECs showed an upregulated expression of endoglin upon injury of the cell monolayer (at 6 and 12 h; Online Resource Supplemental Fig. 1). By contrast, similar wound healing assays in UASMCs did not affect endoglin levels, whereas bmMPCs displayed only a slight increase at 24 h (Online Resource Supplemental Fig. 1). Upon treatment with specific siRNA (Fig. 1b, right panels), endoglin expression was silenced in HUVECs (20.9-fold reduction; MFI, 23.06 vs. 1.1), HAECs (5.1-fold reduction; MFI, 3.07 vs. 0.6) and UASMCs (8.3-fold reduction; MFI, 15.8 vs. 1.9). As a negative control, transfection with two different scrambled siRNA did not affect endoglin expression levels compared to untreated cells. No significant differences in endoglin silencing efficiency were observed between early passages (p2–4) vs. late passages (p8–10) of these cultured cells. The vast majority of HUVECs cotransfected with GFP and endoglin siRNA expressed both GFP and downregulated endoglin. It has been shown that endoglin plays a crucial role in in vitro and in vivo angiogenesis [21, 30, 50]. To assess whether the endoglin suppression achieved in ECs was associated with a decreased endoglin-dependent function, tubulogenesis (a hallmark function of ECs) assays in matrigel were carried out. Figure 1c shows that silencing of endoglin with siRNA in HUVECs and HAECs leads to an impaired tube formation respect to cells transfected with scrambled siRNA or untreated cells. As expected, addition of SolEng (1 μg/mL) also interfered with this angiogenesis assay, in agreement with previous reports [24, 34], suggesting that SolEng counteracts the normal function of membrane bound endoglin. The formation of blood vessels is initiated by endothelial tubulogenesis, followed by the recruitment of mural cells. To assess the role of endoglin in the interaction between endothelial cells and mural cells, these two cell populations were used in 3D tubulogenesis assays. To visualize the cellular adhesion, UASMCs were labeled with GFP and then cocultured with HUVECs at a 1:4 ratio. Adhesion of mural cells to endothelial cells was clearly inhibited by SolEng as well as by suppressing endoglin expression in HUVECs, in UASMCs or simultaneously in both cell types (Fig. 2a, b). High magnification photographs clearly showed binding of green-labeled UASMCs to EC tubules, but not to the surrounding extracellular matrix (ECM) excluding a relevant contribution of cell adhesion to the substrate (Online Resource Supplemental Fig. 2A). Tubulogenesis studies in matrigel also showed that SolEng was able to abrogate the adhesion of UASMCs to HAECs (Online Resource Supplemental Fig. 2A). These data suggest that endoglin is involved in the interaction between endothelial cells and mural cells. Interestingly, silencing of endoglin in mural cells led to an important reduction in VSMC adhesion to ECs (from 32 to 2.2 %). Endoglin expression in resting VSMCs in vivo is relatively low, while it is upregulated under inflammatory conditions or upon in vitro culture [51–53], suggesting that our in vitro model somehow mimics endoglin function in inflammation. Overall, suppression of membrane endoglin, or addition of SolEng, induced a change in the morphology of ECs and VSMCs making them more round, further supporting the role of endoglin in cell adhesion (Fig. 2c, d). Adhesion of CSFE-labeled VSMCs was clearly abolished in the presence of SolEng. Furthermore, adhesion experiments of UASMCs to HAECs, representative ECs typically enveloped by VSMCs, clearly showed the involvement of endoglin in the interaction between ECs and mural cells using SolEng or endoglin siRNA (Online Resource Supplemental Fig. 3). Together, these data suggest that endoglin is involved in the interaction between endothelial and mural cells.

### Integrins are involved in the interaction between endothelial cells and mural cells

**Fig. 3:**
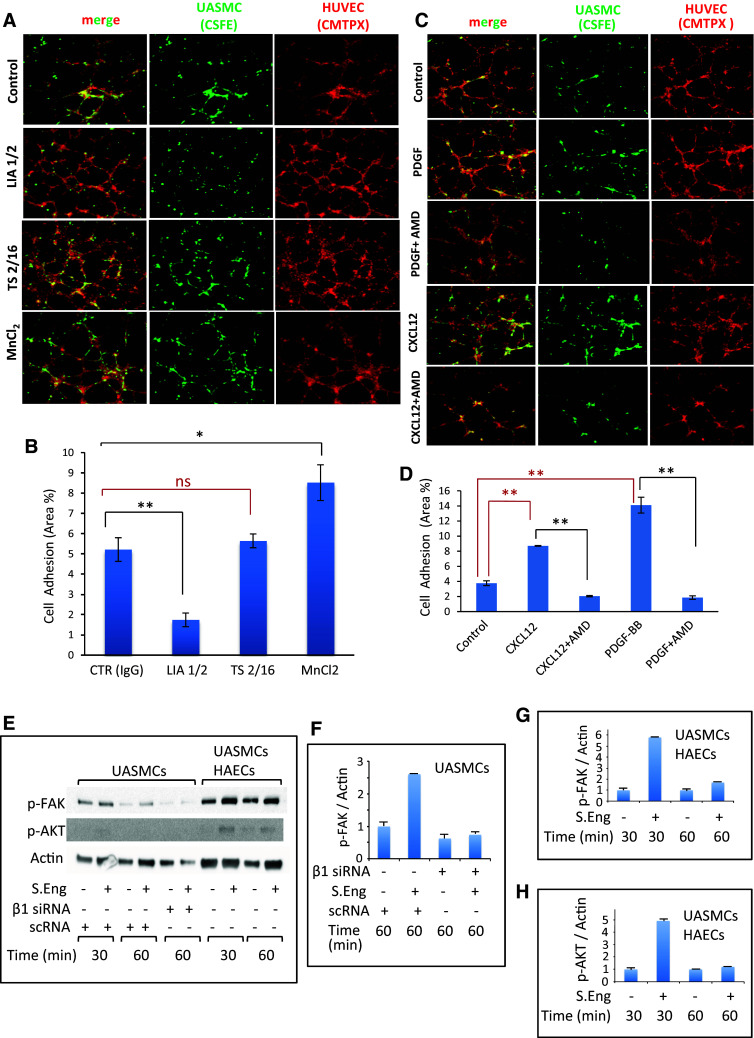
Role of integrins in adhesion between VSMCs and ECs. UASMCs were labeled with CSFE (*green*) and HUVECs were labeled with CMTPX (*red*). Then, UASMCs were cocultured with HUVECs at a 1:4 ratio in matrigel to analyze mural cell adhesion to ECs. **a**, **c** Representative photographs of confocal microscopy analysis. **a** Cells treated with a control mAb IgG2b (CTR) and cells incubated with the inhibitory anti-β1 integrins mAb LIA1/2, the anti-β1 integrins mAb TS2/16 or the general integrins activator MnCl_2_. **c** Untreated cells (control) and cells incubated with the pericyte recruiter PDGF-BB or the integrins activator CXCL12 either in the absence or in the presence of the chemokine receptor (CXCR4) inhibitor AMD3100 (AMD). **b**, **d** Quantification of UASMCs binding to HUVECs from **a** and **c**, respectively. The intensity profile was measured using fluorescence confocal microscopy (SP5, Leica). The mean area in percentage, representing mural cell adhesion measured in different fields, is indicated. Histograms in **b** and **d** represent the mean of four and five independent experiments, respectively. The statistical significance respect to control value (CTR) is indicated. *p < 0.05; **p < 0.005; *ns* not significant. **e**–**h** Effect of soluble endoglin on Akt and FAK phosphorylation. UASMCs were transfected or not with β1-integrin siRNA or scramble siRNA (scRNA). Cultures of UASMCs or cocultures of UASMCs and HAECs were incubated in the absence or presence of 1 μg/mL SolEng. At the times indicated, adherent cells were lysed and proteins were subjected to SDS-PAGE, followed by immunodetection with anti-p-FAK (Tyr925), anti-pAkt (Ser473) or anti-actin antibodies (**e**). Histograms represent the p-FAK/actin ratio in UASMCs (**f**), p-FAK/actin ratio in UASMCs/HAECs (**g**) and the p-Akt/actin ratio in UASMCs/HAECs (**h**). This is a representative experiment of five different ones

**Fig. 4:**
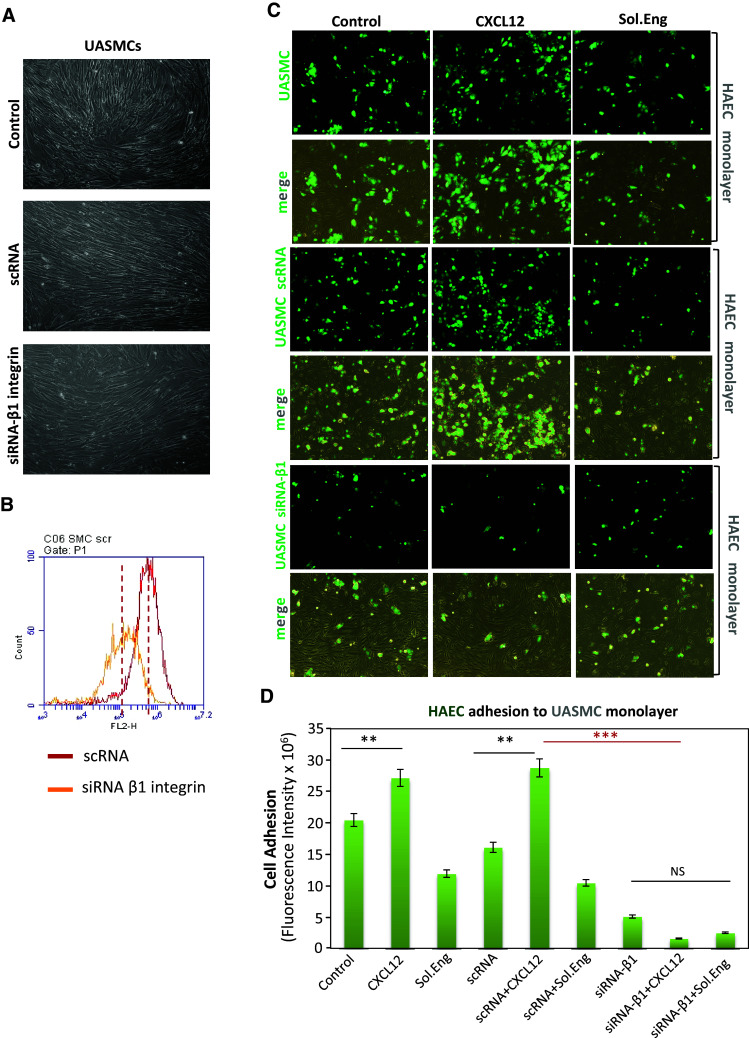
Silencing of β1-integrin in UASMCs. Primary cultures of UASMCs were transfected with beta1 integrin specific siRNA (siRNA-β1) or scrambled siRNA (scRNA). Transfected UASMCs were morphologically (**a**), phenotypically (**b**) and functionally (**c**, **d**) analyzed. **a** Untreated UASMCs (control) and cells transfected scRNA display the same morphology with slight changes respect to cells transfected with siRNA-β1, likely due to the β1 integrin role in cell adhesion. **b** Immunofluorescence flow cytometry with anti-CD29 (anti-β1 integrin) antibodies show a downregulation of β1 integrin (76 %) in UASMCs transfected with specific siRNA vs. cells transfected with scrambled siRNA. **c** Cell–cell adhesion assays. Confluent monolayers of HAECs were incubated with UASMCs, previously labeled with CFSE, in the absence (control) or presence of 1 μg/mL SolEng or 100 ng/mL CXCL12, as indicated. After 1 h incubation, wells were washed and the cells were visualized by confocal microscopy. **d** Binding of HAECs to UASMCs in **c** was quantified by measuring the fluorescence intensity using Image J and Histolab™ (Microvision) software. A representative experiment out of four made in triplicate with similar results is shown (**p < 0.005; ***p < 0.001)

Because endothelial endoglin is a ligand for β1 integrins [16] and VSMCs, pericytes and podocytes express β1 integrins [13, 19], we next assessed whether these receptors were involved in adhesion of mural cells to endothelial cells, using specific antibodies and activators. Thus, adhesion of UASMCs to HUVECs in matrigel plates was clearly inhibited in the presence of the blocking anti-β1 mAb LIA1/2 (Fig. 3a, b), whereas the non-inhibitory anti-β1 mAb TS2/16, did not affect adhesion. By contrast, MnCl_2_, a global activator of integrin activity, induced an enhanced cell adhesion between ECs and VSMCs. Furthermore, the chemokine CXCL12 that activates integrins, allowing their binding to endoglin [16], clearly enhanced adhesion of UASMCs to HUVECs in matrigel (Fig. 3c, d). The effect of CXCL12 is likely mediated by the chemokine specific receptor CXCR4, whose expression could be detected at the cell surface of UASMCs (Online Resource Supplemental Fig. 2B, C). Interestingly, PDGF-BB, a well known mural cell recruiter to vessels, whose expression is induced by CXCL12 [8], markedly increased the adhesion between UASMCs and HUVECs. In addition, CXCL12- and PDGF-dependent cell adhesion was abolished in the presence of AMD3100, an inhibitor of the CXCL12/CXCR4 pathway (Fig. 3c, d), supporting the critical role of CXCL12-dependent integrin activation in these assays. Adhesion studies of UASMCs to HAECs also showed an enhanced cellular adhesion in the presence of CXCL12 (Online Resource Supplemental Fig. 2A). Adhesion by means of β1-integrins is known to induce phosphorylation of protein kinases Akt and FAK. Importantly, addition of SolEng to UASMCs alone or in co-culture with HAECs led to an increased phosphorylation of Akt and FAK, an effect that was inhibited by siRNA-mediated depletion of β1-integrin (Fig. 3e–h). However, addition of SolEng to individual cultures of HAECs did not induce phosphorylation of either Akt or FAK, suggesting that SolEng specifically targets integrins on UASMCs. Furthermore, basal and CXCL12-induced adhesion between UASMCs to HAECs were abolished upon siRNA-mediated knockdown of β1-integrin in UASMCs (Fig. 4).

### Role of the RGD motif of endoglin in the adhesion between ECs and VSMCs

**Fig. 5:**
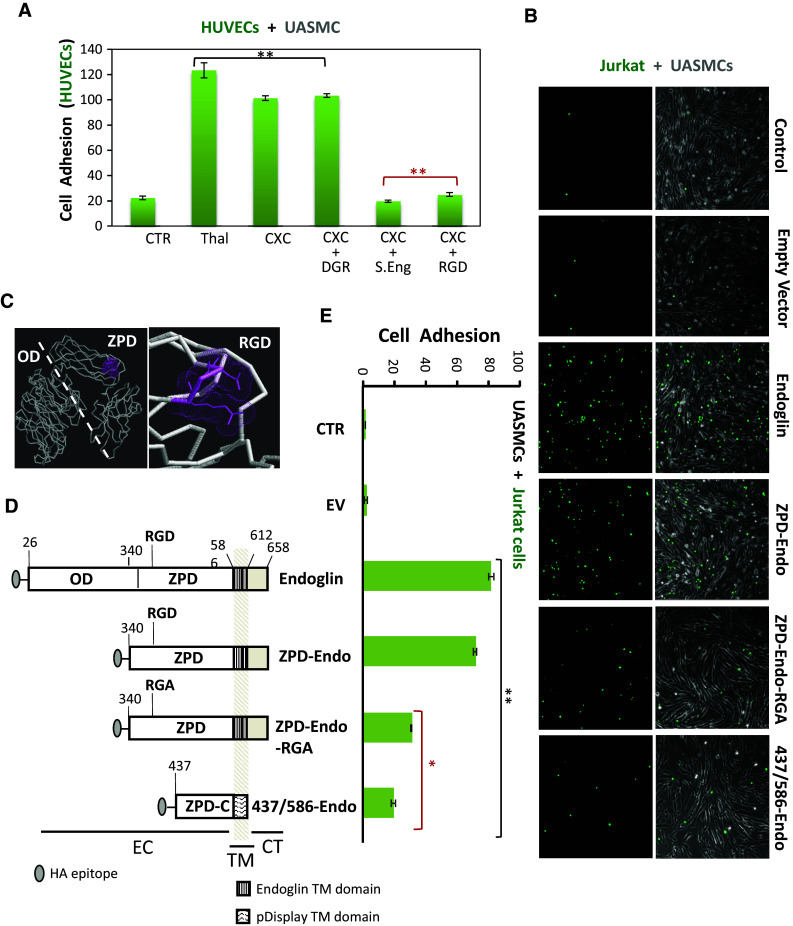
Involvement of the endoglin RGD motif in adhesion between VSMCs and ECs. **a** HUVECs, labeled with CSFE (*green*), were incubated with the UASMC monolayers for 1 h at 37 °C with/without thalidomide, CXCL12 (CXC), SolEng (S.Eng), RGD peptide or DGR peptide, as indicated. Bound CSFE-labeled cells were lysed and quantified by Varioskan plate reader. The average of four different experiments in duplicate is shown. Statistical significances vs. control cells (**p < 0.005) or vs. CXCL12-treated cells (**p < 0.005; *red asterisks*) are indicated. **b**–**e** Adhesion of different deletion constructs of endoglin to VSMCs. **c** Atomic model for endoglin and location of RGD motif. The extracellular domain of endoglin contains an orphan domain (OD) and a juxtamembrane zona pellucida domain (ZPD) that includes an RGD motif (in *magenta*). The *dashed line* separates the OD from the ZPD. **d** Generation of different truncated forms of endoglin. *Numbers* indicate the amino acid of endoglin (starting at the N terminus) that limit the corresponding fragment. The position of extracellular (EC), transmembrane (TM), and cytoplasmic (CT) domains, is indicated. All of the constructs contain the leader sequence of the IgGκ and the HA epitope at the N terminus (from the pDisplay vector), and construct 437/586-Endo encode the transmembrane domain of the pDisplay vector. The OD encompasses amino acid residues 26–359, whereas the ZP domain is contained within the fragment 360–586. The ZPD-C (residues 437–586) is a sub-domain of ZPD. The presence of the RGD motif (residues 399–401) and its mutant version (RGA) is indicated. **b**, **e** Adhesion assays of Jurkat cells to VSMCs. Jurkat cells were nucleofected with endoglin, ZPD-Endo, ZPD-Endo-RGA or 437/586-Endo constructs together with GFP. As negative controls, cells were nucleofected with GFP in the absence (CTR) or in the presence of the empty vector (EV). After nucleofection, Jurkat cells were added to the UASMC monolayers for 1 h at 37 °C. Bound GFP expressing cells were lysed and quantified by Varioskan plate reader (**e**). A representative experiment out of three made in triplicate with similar results is shown. Statistical significances vs. control cells (**p < 0.005) or cells expressing full length endoglin (*p < 0.05; *red asterisks*) are indicated

To investigate the possible involvement of endoglin in the CXCL12-dependent integrin activation for cell adhesion, additional experiments in the presence of SolEng and RGD peptides were carried out. In agreement with the matrigel assays of Fig. 3, adhesion of HUVECs to UASMCs monolayers was markedly enhanced by addition of CXCL12 and this increase was abolished by the presence of SolEng (Fig. 5a). Of note, SolEng has been previously shown to specifically inhibit the interaction between endothelial endoglin and leukocyte integrins [16]. As a positive control, incubation with thalidomide increased HUVECs adhesion to mural cells in agreement with a previous report [32]. Similar results as in Fig. 5a were obtained with the reverse experiment, i.e., adhesion of VSMCs to ECs monolayer (Fig. 2c, d). Next, we investigated the possible involvement of endoglin’s RGD as a recognition motif of β1 integrins in VSMCs. The RGD motif is contained within the juxtamembrane ZP domain of endoglin (Fig. 5c) in an accessible hydrophilic region of the protein [22]. As shown in Fig. 5a, addition of the human endoglin-derived DRGDK peptide inhibited the adhesion of HUVECs to UASMCs monolayers, whereas the negative control peptide SDGRG did not affect cell adhesion, suggesting that the RGD motif of endoglin is involved in the binding to β1 integrins in VSMCs. To further investigate the adhesive capacity of the RGD motif of endoglin, different deletion and mutant constructs were used in presence of CXCL12 (Fig. 5b, d, e). The activity in cell adhesion of these constructs was assayed by transfecting them into Jurkat cells followed by incubation with an UASMC monolayers (Fig. 5e, f). Jurkat cells were selected because they do not express endogenous endoglin, grow in suspension and show a negligible adhesion to UASMCs under basal conditions. Upon cell transfection, recombinant endoglin proteins were easily detected by western blot and immunofluorescence flow cytometry analyses (Online Resource Supplemental Fig. 4). Expression of full length endoglin or a truncated endoglin construct with the RGD-containing ZP domain (ZPD-Endo) dramatically increased adhesion of Jurkat cells to UASMCs (Fig. 5b, e). By contrast, expression of the ZP domain with an inactive mutant version of the RGD motif (ZPD-Endo-RGA) or an RGD-less shorter construct containing the carboxy-terminal ZP subdomain (437/586-Endo) showed a marked reduction (62 and 76 %, respectively), in the adhesion of Jurkat cells to UASMCs respect to cells transfected with the endoglin full length construct. Together, these results suggest that the RGD motif of membrane endoglin is involved, via integrins, in the binding to VSMCs.

### Binding of SolEng to VSMCs and its in vivo effect

**Fig. 6:**
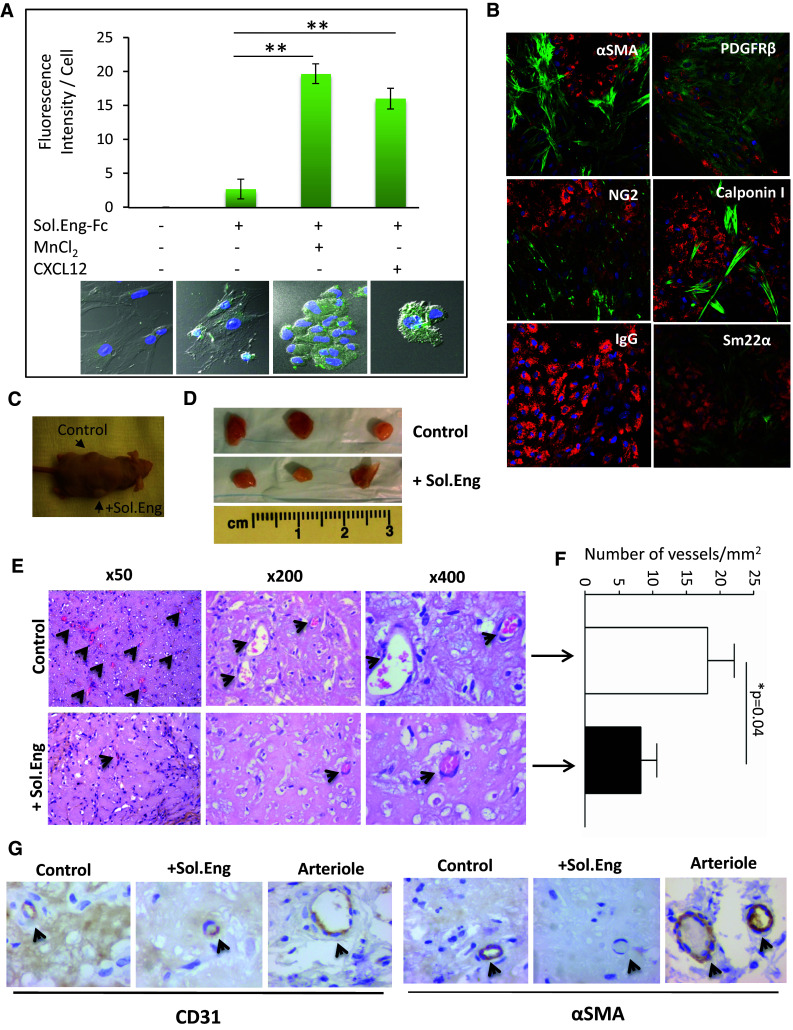
Soluble endoglin binds to VSMCs and inhibits angiogenesis in vivo. **a** Binding of soluble endoglin to VSMCs. Exponentially growing UASMCs were incubated with a chimeric protein containing the extracellular domain of endoglin fused to the Fc fragment of IgG (Sol.Eng-Fc), and the integrins’ activators CXCL12 and MnCl_2_, as indicated. After incubation with FITC-labeled anti-human IgG, cells were fixed, counterstained with DAPI and analyzed by confocal microscopy (*lower panel*). The fluorescence intensity was measured using confocal microscopy (SP5, Leica) and represented as mean values per cell. A representative experiment out of three made in triplicate with similar results is shown. The statistical significance is indicated (**p < 0.005). **b** In vitro 7 days co-culture of bmMPC and ECFC. bmMPC differentiate into VSMC/pericytes as evidenced by the positivity for the antibodies specific for αSMA, PDGFRβ, NG2, Calponin I and Sm22α (*green stain*). A negative control antibody (IgG) was also used. Endothelial cells (ECFC) are stained in *red* using an antibody against von Willebrand factor. **c**–**f** Effect of soluble endoglin on in vivo angiogenesis. **c** Nude mice (*n* = 3) were injected with the coculture of bmMPC + ECFC in matrigel either in the absence (control) or in the presence of soluble endoglin (Sol.Eng; 5 μg/mL/plug). **d** After 1 week, plugs were extracted from the animals. **e** Plug sections were stained with hematoxylin and eosin. *Arrowheads* indicate the presence of vascular structures containing erythrocytes. **f** Quantification of the vessels. The average of three different experiments made in triplicate is shown (N nude mice = 9; *n* = 9 plugs for each condition). The plugs treated with SolEng display a lower number of vessels compared to controls (*p < 0.04). **g** Immunohistochemistry of plug sections. *Arrowheads* indicate the staining of vascular endothelium with anti-CD31 antibodies and mural cells with anti-αSMA. Staining of αSMA is almost absent in plugs treated with soluble endoglin. A positive staining of arterioles from the neighboring tissue is included as an internal control

**Fig. 7:**
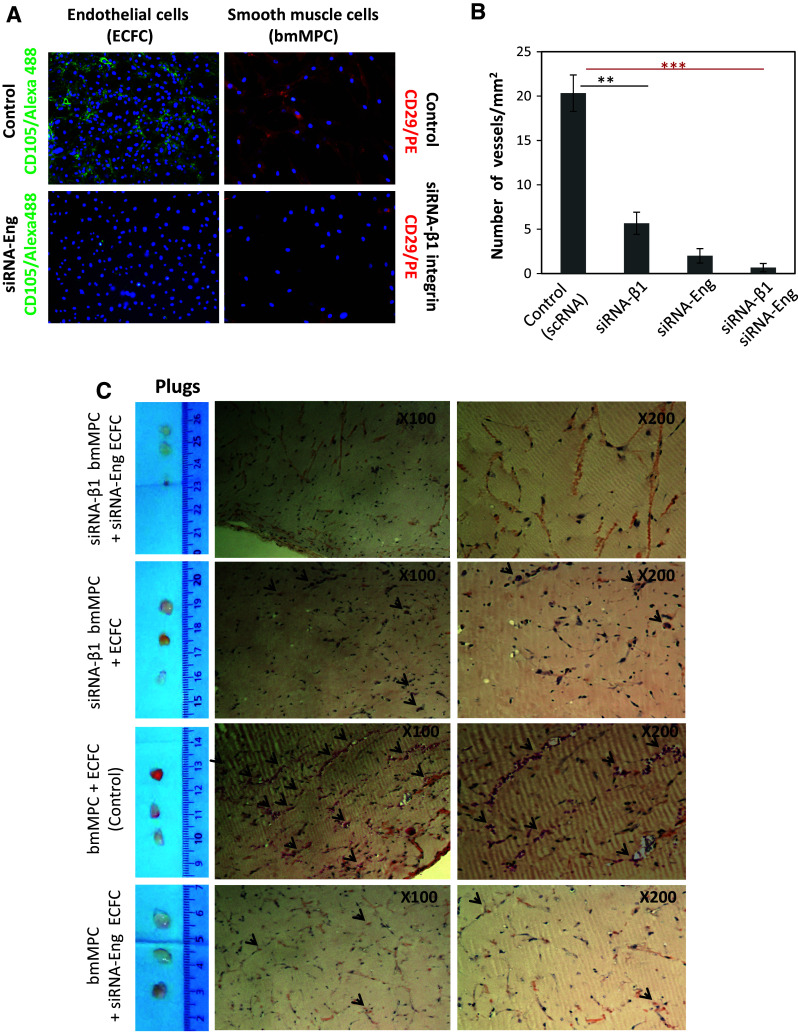
Effect of silencing endoglin and β1 integrin in angiogenesis assays in vivo. **a** Suppression of β1 integrin and endoglin using specific siRNA was carried out in cultured bmMPC and ECFC, respectively. Endothelial cells (ECFC) or smooth muscle cells (bmMPC9) were stained with anti-CD105 (endoglin) in *green* or anti-CD29 (β1 integrin) in *red*, respectively. **b** Quantification of the vessels. The average of two different experiments performed in triplicate is shown. The individual or combined suppression of endoglin or β1 integrin in bmMPC or ECFC, respectively, leads to a markedly reduced number of vessels compared to controls (**p < 0.01; ***p < 0.005). **c** Four groups of nude mice (*n* = 3 each) were injected with the coculture of bmMPC + ECFC in matrigel either in the absence (scrambled siRNA; control) or in the presence of siRNA-mediated suppression, as indicated. After 1 week, plugs were extracted from the animals and stained with hematoxylin and eosin. *Arrowheads* indicate the presence of vascular structures containing erythrocytes

The experiments shown in Figs. 2, 3 and 4 indicate that SolEng can inhibit the integrin-mediated interaction between ECs and mural cells. We then wondered whether this inhibitory effect involved a stable binding of SolEng to VSMCs. To assess this binding, a chimeric protein containing the extracellular domain of endoglin fused to the human Fc fragment [47] was used. As shown in Fig. 6a, binding of SolEng to untreated VSMCs was markedly enhanced in the presence of the integrin activators CXCL12 and MnCl_2_, suggesting that SolEng stably binds to activated integrins on the cell surface (Online Resource Supplemental Material, Video #1). Of note, SolEng-treated cells slightly changed their morphology and clustered (Fig. 6a), a phenomenon likely involving integrins-mediated cell interactions with the ECM proteins. To assess the effect of SolEng in vivo, a well-established differentiation protocol involving the co-culture of human bone marrow mesenchymal precursor cells (bmMPC) and human cord blood-derived endothelial colony forming cells (ECFC) was used [43]. Upon co-culture for 7 days, ECFC induce bmMPC differentiation into VSMCs/pericytes and specific markers were detected, including αSMA, PDGFRβ, NG2, Calponin I and Sm22α (Fig. 6b). As expected, ECs also expressed the endothelial-specific von Willebrand factor (Fig. 6b), as well as endoglin (Online Resource Supplemental Fig. 5A). Then, nude mice were injected with these co-cultures in matrigel in the absence or in the presence of SolEng and after 1 week, the plugs were analyzed (Fig. 6c, d). Hematoxylin and eosin staining of the plugs revealed that SolEng inhibits the angiogenic process (Fig. 6e, f), suggesting again that SolEng impairs the assembly of ECs and mural cells, thus interfering with the tubulogenesis process (Fig. 6g; Online Resource Supplemental Fig. 5B). The involvement of membrane bound endoglin and β1-integrin in the in vivo model of tubulogenesis was further supported by siRNA-mediated suppression experiments (Fig. 7). The expression of endoglin or β1-integrin in ECFC or bmMPC, respectively, was knockdown in vitro by siRNA (Fig. 7a), leading to a markedly reduced number of vessels in vivo, compared to controls (Fig. 7b, c). These results suggest that endoglin in ECFC and β1-integrin in bmMPC are necessary for the proper assembly of ECs and mural cells in vivo.

### Endoglin deficiency leads to an increased pericyte-dependent EC permeability

**Fig. 8:**
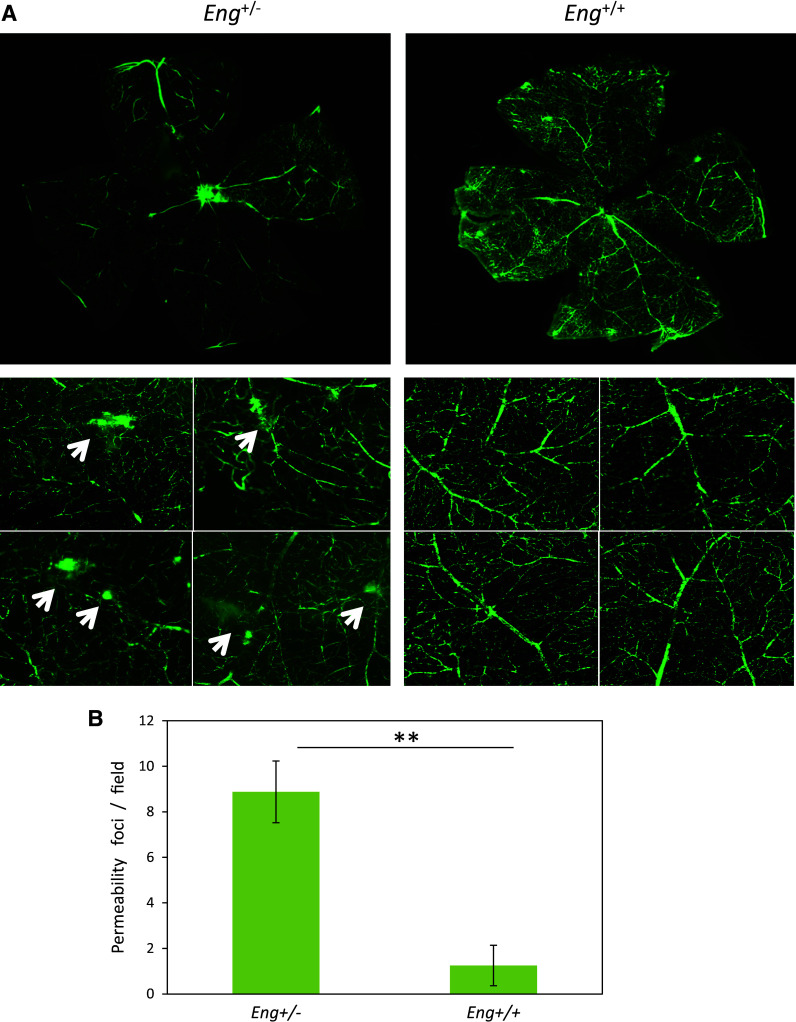
Increased vascular permeability in *Eng* ^+/−^ mice. *Eng* ^+/−^ and *Eng* ^+/+^ mice were perfused through the jugular vein with FITC-dextran. After 2 h, animals were killed and eyes were removed and dissected. Nineteen neuroretinas from ten different animals (six *Eng* ^+/+^ and four *Eng* ^+/−^ mice) were isolated and mounted with antifade solution. The *green* fluorescent labeling of the retinas was visualized using a fluorescence microscope (Axiovert 200M, Zeiss). **a** Representative photographs from three *Eng* ^+/+^ and three *Eng* ^+/−^ mice, are shown. An increased number of permeability foci and diffuse *green* fluorescence background is observed in *Eng* ^+/−^ mice (*arrows*) compared with *Eng* ^+/+^. **b** Quantification of permeability spots (**p = 0.01)

Pericytes embracing the endothelium regulate the blood retinal barrier. Vascular permeability studies were carried out in endoglin heterozygous mice (*Eng* ^+*/*−^), as a model of HHT1 [29]. Measured by passage of fluorescent dextran, the retinas from *Eng* ^+*/*−^ mice (*n* = 8) displayed a higher number of permeabilization foci (Fig. 8, arrows) compared to *Eng* ^+*/*+^ retinas (*n* = 10), suggesting a destabilization of the endothelial barrier function due to endoglin haploinsufficiency (Fig. 8a, b). These results support our hypothesis that endoglin is involved in the adhesion between ECs and pericytes and consequently in vessel stabilization. Of note, the increased vascular permeability in the neuroretina (Fig. 8) is compatible with the decreased inflammation-induced transendothelial migration of leukocytes [16] observed in *Eng* ^+/−^ mice, as well as with the leukocyte infiltration detected in glomeruli of *Sol.Eng* mice [35]. In this line, vascular permeability is a passive process that allows plasma to pass through the endothelium, whereas the leukocyte transmigration is an active process that involves the stimulation of leukocytes by an inflammatory stimulus, followed by different sequential highly regulated steps, including rolling, activation, firm adhesion and transendothelial migration (diapedesis), respectively.

### Effect of SolEng in podocyturia and its involvement in preeclampsia

**Fig. 9:**
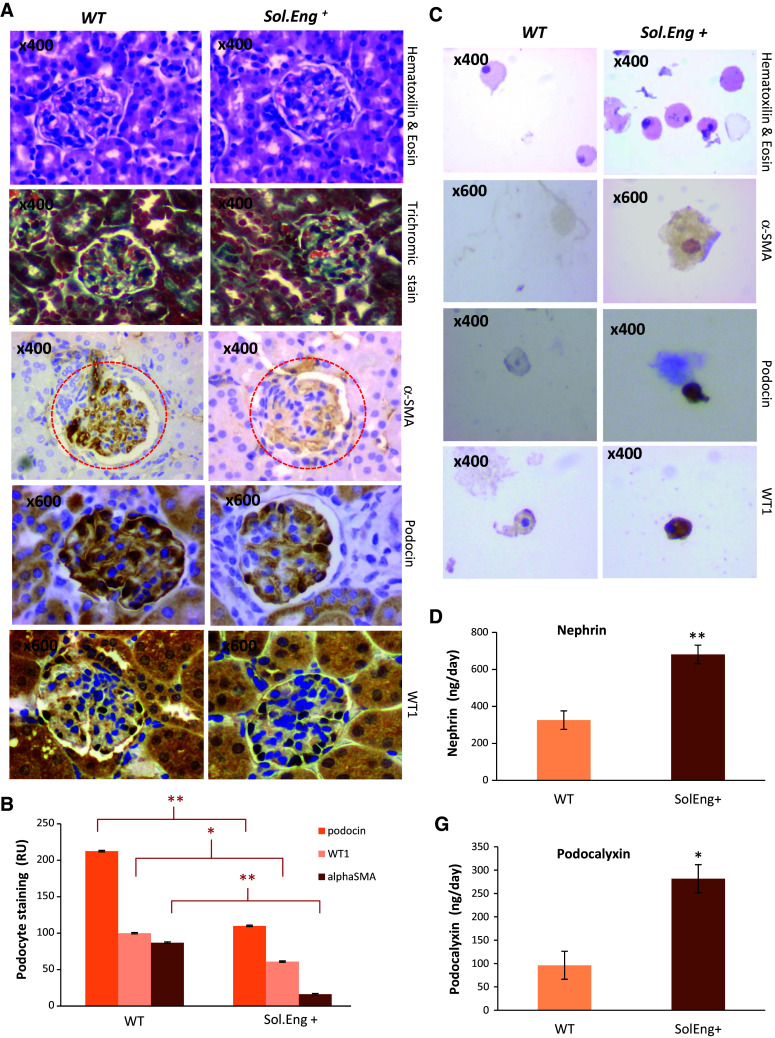
Analysis of podocytes in the kidney from *Sol.Eng* ^+^ mice. **a** Comparison between glomeruli of *Sol.Eng* ^+^ mice (*n* = 17) and controls (*n* = 17). The overall structure of glomeruli in WT and transgenic animals is similar, as evidenced by the hematoxylin and eosin and trichromic staining, although a certain degree of leukocyte infiltration was observed in *Sol.Eng* ^+^ mice. Immunostaining with anti-αSMA, anti-podocin and anti-WT1 shows a positive signal in podocytes from wild type animals and a reduced staining in the *Sol.Eng* ^+^ glomeruli. Representative tissue sections are shown. **b** The immunostaining of αSMA, podocin and WT1 shown in **a** was quantified using the Image J software. Relative units (RU) of podocyte staining from four different experiments are represented in the *vertical axis*. **c** Thinprep on urine from *Sol.Eng* ^+^ mice (*n* = 5) and controls (*n* = 5). Hematoxylin and eosin staining shows the presence of cells in urine. Staining with anti-α-SMA, anti-podocin and anti-WT1 indicates the presence of podocytes in the urine of *Sol.Eng* ^+^ mice. **d**, **g** Protein levels of nephrin and podocalyxin were measured in the urine of WT (*n* = 5) and *Sol.Eng* ^+^ (*n* = 5) mice using an ELISA kit

In the kidney, podocytes are bound to glomerular basement membrane (GBM) through several β1 integrins [19]. Because podocyturia is present in patients with preeclampsia, a disease associated with elevated levels of SolEng in serum [37], we postulated that SolEng could contribute to podocyturia by detaching podocytes from capillaries. To test this hypothesis, transgenic mice expressing constitutively high levels of human SolEng (*Sol.Eng* ^+^), which are a model of preeclampsia [35], were used. The transgenic SolEng was easily detected in the urine of *Sol.Eng* ^+^ mice (>15 ng/mL in all animals; *n* = 9), whereas it was almost undetectable in wild type animals (<0.12 ng/mL; *n* = 4). Staining of glomeruli with anti-αSMA and the specific markers podocin and Willms Tumor (WT1) revealed that the number of podocytes is reduced in *Sol.Eng* ^+^ compared to wild type mice (Fig. 9a, b). In some cases, a dramatic loss of αSMA and podocin in *Sol.Eng* ^+^ mice was observed (Online Resource Supplemental Fig. 6A, B). Compatible with these findings, hematoxylin and eosin staining shows an increased number of cells in the urine of *Sol.Eng* ^+^ vs. WT mice (Fig. 9c, top panels) and staining with anti-αSMA, anti-podocin and anti-WT1 indicates the presence of podocytes in the urine of *Sol.Eng* ^+^ mice (Fig. 9c). Furthermore, the levels of nephrin and podocalyxin, two markers of podocyturia, were significantly higher in the urine of *Sol.Eng* ^+^ vs. WT mice (Fig. 9d). All these data suggest the presence of podocyturia in *Sol.Eng* ^+^ mice. To support the hypothesis that SolEng induces the detachment of podocytes and allows the filtration of large molecules in *Sol.Eng* ^+^ mice, we analyzed the presence of endoglin in the glomeruli by IHC and in the urine by western blot. As shown in Online Resource Supplemental Fig. 6C, D, a marked endoglin staining was found in the glomeruli of *SolEng* ^+^ mice and SolEng was detected by western blot analysis in the urine of *SolEng* ^+^ mice, while it was absent in WT animals. Taken together, these results suggest that SolEng is able to detach podocytes from glomerular capillaries giving rise to a defect in permeability and vessel stabilization.

## Discussion

**Fig. 10:**
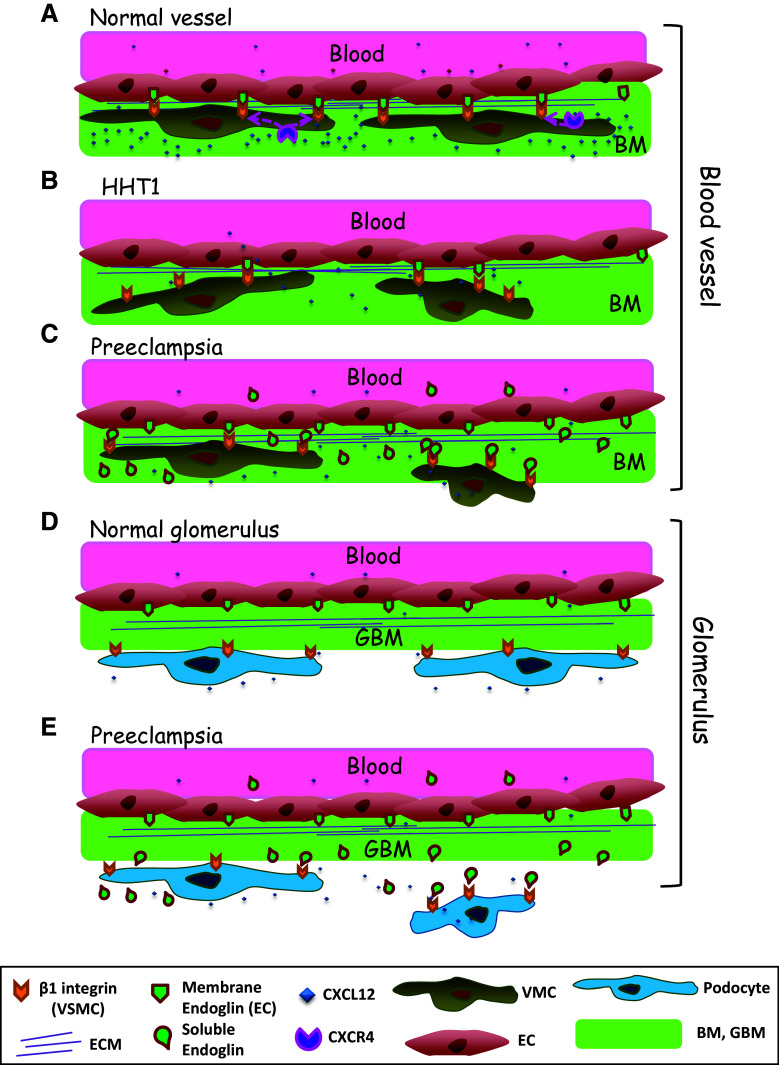
Schematic diagram showing the role of endoglin in integrin-mediated cell adhesion between ECs and mural cells/podocytes. **a**–**c** Blood vessels. **a** A normal blood vessel with an endothelial monolayer facing the lumen surrounded by vascular mural cells (VMCs) and ECM proteins. ECs and vascular mural cells share a common basal membrane (BM). During vascular development and stabilization, binding of the homeostatic chemokine CXCL12 to its receptor CXCR4 leads to activation of β1-integrins in VMCs. Then, endothelial endoglin binds to β1-integrins on VMCs. **b** In HHT1, endoglin haploinsufficiency leads to a decreased binding of endothelial endoglin to β1-integrins in VMCs. **c** In preeclampsia, soluble endoglin competes with membrane bound endoglin for the binding to β1-integrins in VMCs. **d**, **e** Kidney glomerulus. **d** A normal glomerulus showing pericytes bound to the glomerular basal membrane (GBM) through their surface integrins. **e** In preeclampsia, soluble endoglin competes with GBM for the binding to surface integrins in podocytes. The presence of endothelial endoglin in the lumen of the vessel and the existence of other adhesion molecules have been omitted for simplification

During blood vessel assembly and maturation, ECs and the vessel basal membrane recruit different types of mural cells, including VSMCs and pericytes. Our data suggest that endothelial endoglin plays a role in the recruitment of these mural cells and in vessel stabilization. In agreement with this conclusion, previous reports have shown that in yolk sacs from endoglin KO mice, the levels of αSMA are strikingly decreased because of the failure of vascular VSMCs to differentiate and associate with ECs, so that blood vessels become fragile and dilated [28, 54]. Of note, these latter features are the hallmark of vascular lesions in HHT, a disease originated by heterozygous mutations in the endoglin gene [27]. In spite of the experimental evidences supporting the role of endoglin in the interplay between ECs and VSMCs, the molecular bases for the endoglin involvement are not completely understood. Endoglin is expressed at high levels in ECs undergoing active proliferation or angiogenesis [21], while its levels in VSMCs are very low or undetectable in normal conditions, but they can be increased upon in vitro culture, during atherosclerosis or vascular injury [51–53]. Additional differences between cultured ECs and vascular mural cells, regarding basal and stimulated expression levels of endoglin, have been observed (Fig. 1b; Online Resource Supplemental Fig. 1). While the role of endoglin in vascular mural cells deserves an independent analysis, we have focused our study on the role of endothelial endoglin in the adhesion between ECs and vascular mural cells (Fig. 10a–c). Silencing endoglin in ECs (HUVECs and HAECs) results not only in inhibition of tube formation in 3D matrigel assays, but also in a severely compromised attachment of UASMC to ECs in 3D co-cultures. These data indicate a critical role for endothelial endoglin in the interaction between ECs and VSMCs. Moreover, our results suggest the involvement of the RGD motif of endoglin in its binding to integrins. Members of the RGD family of related peptides have in common the presence of an acidic residue (D or E) that is critically involved in coordinating a Mg^2+^ cation bound to the MIDAS motif in integrin β subunits [55] and this acidic residue is conserved among mammalian endoglins [16]. Of note, the homologous sequence of the human RGD in mouse endoglin is TDD a motif that has also been shown to be involved in integrin-mediated cell adhesion and is present in the disintegrin domain of murine ADAM-15 [16, 56].

The specific integrin involved in the binding to endoglin is likely integrin α5β1, which specifically recognizes the RGD motif in extracellular matrix proteins and it is expressed by VSMCs, regulating their adhesion and recruitment to blood vessels [13]. In fact, the adhesion of UASMCs to ECs is reduced by suppressing β1-integrin using specific siRNA and by the β1-integrin inhibitory antibody LIA1/2. Conversely, incubation with CXCL12, an activator of integrins markedly enhanced adhesion of VSMCs to ECs and this effect was abolished in the presence of SolEng or an endoglin-derived peptide containing the RGD motif. Interestingly, pretreatment of UASMCs with the proinflammatory chemokine CXCL12 was sufficient to enhance adhesion to ECs, suggesting that activation of VSMC integrins promote their binding to endothelial endoglin. While VSMCs are not typically considered targets of vascular inflammation [57], endoglin expression in endothelial cells promotes smooth muscle precursor cell recruitment and vessel maturation via regulation of the CXCL12/CXCR4 signaling axis [58]. In this line, our results point out the potential mural cells responsiveness in an inflammatory setting, at least, regarding the endoglin/integrin-mediated responses. The effect on vascular function upon the inducible deletion of β1 integrin family members has been previously reported. Thus, the β1-integrin deletion in VSMCs of adult mice leads to catastrophic effects on the vascular system and the animal dyes after 10 days of inducing β1-integrin suppression, suggesting that β1-integrin in VSMCs is involved in vascular function [59]. Moreover, mice lacking the RGD-binding α5 and αv integrins (both partners of β1-integrin) on VSMCs develop interrupted aortic arches, large brachiocephalic/carotid artery aneurysms and cardiac septation defects [60]. These results are in agreement with our tubulogenesis results demonstrating that β1-integrins in bmMPC are necessary for the proper assembly of ECs and mural cells in vivo (Fig. 7). Further support for the involvement of the endoglin RGD motif was obtained from cellular adhesion experiments using endoglin mutant constructs. Thus, the RGD containing ZP domain was identified as the region responsible for endoglin binding to VSMCs. Compatible with these adhesive properties, the ZP domain is a conserved module for polymerization of extracellular proteins present in a large family of ZP proteins [61]. However, these experiments do not rule out an RGD-independent involvement of endoglin in adhesion to VSMCs. Indeed, treatment with MnCl_2_, a general activator of integrins, stimulated adhesion of UASMCs to ECs, suggesting the involvement of other integrin family members as well as non-RGD mediated endoglin interactions with integrins. This interpretation agrees with co-immunoprecipitation experiments between endoglin and the α5 integrin subunit demonstrating that endoglin can bind to this integrin independently of the RGD motif [39].

During angiogenesis, the adhesion between ECs and mural cells is finely regulated by soluble factors, including PDGF-BB. Once released by activated platelets, PDGF-BB induces the recruitment of mural cells to endothelial tubes, although the molecular mechanism of this process is not fully understood. We find that in tubulogenesis assays, the PDGF-dependent adhesion of VSMCs to ECs is abolished by an antagonist of CXCR4, the CXCL12 receptor. Because CXCL12 is an activator of integrins and induces an endoglin-mediated adhesion between ECs and VSMCs (Fig. 5), these data are compatible with a PDGF-induced recruitment of mural cells to endothelial tubes mediated by CXCL12 and endoglin.

The evidences for the role of endoglin in ECs–VSMCs adhesion found in this work are in agreement with the poor coverage of vessels by SMCs and with the arrested endothelial remodeling during development of *Eng* ^−/−^ mice [28]. Moreover, the pericyte-dependent permeability in the retina of endoglin heterozygous mice (*Eng* ^+*/*−^), a model of HHT1, shows an increased leakiness and number of permeability foci compared to wild type animals (Fig. 8). In agreement with this finding, a recent article has shown an increased permeability in endoglin-deficient EC monolayers [62]. By contrast, a milder vascular phenotype has been reported upon endoglin suppression in adult mice. Thus, specific deletion of endoglin in 8- to 10-week-old mice does not lead to markedly reduced mural cell coverage, but to an altered localization within arteries and veins [63]. These results suggest that the role of endoglin is critical during *de novo* cell adhesion-dependent neoangiogenesis, such as during development or inflammation. However, once the initial endoglin-dependent adhesion between ECs and SMCs has been established, other adhesion and receptor molecules reinforce the ECs–SMCs interaction and become the predominant adhesion forces over the endoglin-integrin adhesion. Previous studies support the involvement of the interaction between endoglin and integrins in vascular functions such as vasoconstrictor or vasodilator responses where SMCs play a key role. Thus, adult mice lacking SMC β1-integrin show a profound loss of vasomotor control including the EC-dependent vasodilation to acetylcholine and the VSMC-dependent vasoconstriction to norepinephrine [59]. Also, hypotensive and vasodilatory responses induced by acetylcholine and bradykinin in *Eng* ^+/+^ animals were markedly reduced in *Eng* ^+/−^mice [64]. In addition, an upregulation of cyclooxygenase-2 (COX-2) in the vascular endothelium and increased urinary excretion of prostaglandin E2 in *Eng* ^+/−^ compared with *Eng* ^+*/*+^ mice has been reported [48]. These results support the role of β1-integrin and endoglin in vasomotor function.

SolEng, proteolytically cleaved from the membrane form of endoglin, is present at high levels in a variety of pathophysiological conditions including preeclampsia [21]. Although an anti-angiogenic activity for SolEng has been reported [24, 34], its mechanism of action is poorly understood. We found that SolEng can inhibit the adhesion of VSMCs to ECs as well as induce podocytes detachment in the kidney. Based on these results, we postulate that SolEng binds integrins preventing not only adhesion of vascular mural cells to ECs (competing with endothelial membrane endoglin), but also the adhesion of podocytes to the GBM (Fig. 10).This novel role for endoglin and SolEng in cell adhesion during angiogenesis and vessel stabilization involves the ZP domain via its binding to integrins, but does not exclude an additional regulatory role of the orphan domain, which can bind to TGF-β family members [24, 25]. The effect of SolEng in cell adhesion is supported by the morphological changes observed in its presence (Figs. 2c, 6a), which are likely due to the endoglin interaction with surface integrins. Furthermore, that SolEng stably binds to the surface of VSMCs is clearly illustrated after integrin activation with CXCL12 or MnCl_2_ (Fig. 6a; Video #1 in Online Resource Supplemental material). Noteworthy, SolEng induces vascular permeability and hypertension in vivo [34, 35] and these effects are compatible with the antagonist role of SolEng on the interaction between membrane bound endothelial endoglin and β1-integrins present in mural cells or in podocytes. It should be noted that because β1-integrins can interact with various ECM proteins, interference of these activities by endoglin may also contribute to the functional alterations observed. Based on its modulatory action on vascular function, a deleterious effect of soluble endoglin during in vivo embryogenesis, would be expected. Supporting this view, *SolEng* ^+^ female mice crossed with non-transgenic males led to a marked reduction in the weight of pups and the number of pups per litter, respect to wild type mothers [35].

As a further proof of the major effect of SolEng on β1 integrin-mediated cell adhesion, we have assessed the effect of high levels of SolEng on podocytes, present in glomerular capillaries, which play a key role in regulating glomerular protein filtration. It should be noted that patients with preeclampsia present increased levels of SolEng in plasma and proteinuria [34]. Moreover, podocyturia is a diagnostic marker for preeclampsia amongst high-risk pregnant patients [65], and generally correlates with proteinuria [36, 37]. However, the mechanism responsible for podocyturia in preeclampsia is unknown. Thus, we hypothesized that SolEng could contribute to podocyturia by detaching the glomerular podocytes from capillaries. Experimental support for this hypothesis was found using an animal model of transgenic mice (*Sol.Eng* ^+^ *),* that mimics the high expression of SolEng in preeclampsia [35]. The number of mural cells in kidney non-glomerular blood vessels (small arterioles) was similar between *Sol.Eng* ^+^ and wild type mice, whereas the number of glomerular podocytes was greatly reduced in *Sol.Eng* ^+^ compared to wild type mice. This finding is reinforced by the presence podocytes and SolEng in the urine of *Sol.Eng* ^+^, but not of WT mice (Fig. 9; Online Resource Supplemental Fig. 6D). These results suggest that SolEng may lead to the detachment of podocytes from GBM. Of note, podocytes are exposed to permanent transcapillary filtration pressure and flow and must therefore adhere tightly to the underlying GBM. The major cell–matrix adhesion receptor in podocytes is the integrin α3β1, which connects laminin α5β2γ1 in the GBM through various adaptor proteins to the intracellular actin cytoskeleton. Other cell–matrix adhesion receptors expressed by podocytes include the integrins α2β1 and αvβ3 [19]. We have previously shown that endoglin is a ligand for β1 integrins [16]. Thus, we suggest that, in preeclampsia, SolEng that is continuously being filtered through the glomerular capillaries reaching the Bowman’s capsule, can bind podocytes’ β1 integrins and facilitate their detachment from the GBM (Fig. 10d, e). Accordingly, SolEng would play a key role in podocyturia, a major characteristic of preeclampsia. Also, our data provide a further indication that β1 integrins are critical targets for membrane and SolEng.

Endoglin is a component of the TGF-β receptor complex and TGF-β signaling regulates mural cell differentiation and vessel maturation [10]. Thus, it is possible that the endoglin-dependent functions analyzed here are also regulated by the TGF pathway. Indeed, in yolk sacs from *Eng* ^+*/*−^ mice, TGF-β/ALK5/Smad2 signalling from ECs to adjacent mesothelial cells is defective, leading to the failure of vSMCs to differentiate and associate with ECs so that blood vessels are fragile and become dilated [54]. In addition, inhibition of TGF-β signaling by systemic expression of soluble endoglin in adult *Eng* ^+*/*+^ mice, results in reduced levels of phospho-Smad2 in the retina, decreased retinal function, impaired perfusion of the inner retinal vasculature, loss of capillary integrity and breakdown of the blood–retinal barrier [66]. These results are in agreement with our findings of membrane and soluble endoglin and suggest a complex and redundant regulation of vessel maturation.

In summary, we describe here a novel role of endoglin and its soluble form (SolEng) in cellular adhesion involving mural cells/podocytes that may help to better understand the function of endoglin in angiogenesis as well as in several pathophysiological processes, such as preeclampsia or HHT1.

### Electronic supplementary material

Below is the link to the electronic supplementary material.

Supplementary material 1 (PDF 15639 kb)

Supplementary material 2 (MOV 360 kb)

## Abbreviations

bmMPC: Bone marrow-derived mesenchymal progenitor cell
EC: Endothelial cell
ECFC: Endothelial colony-forming cell
Eng: Endoglin
FCS: Fetal calf serum
GBM: Glomerular basement membrane
GFP: Green fluorescent protein
HHT1: Hereditary Hemorrhagic Telangectasia type 1
HAEC: Human artery endothelial cells
HUVEC: Human umbilical vein endothelial cell
MFI: Mean fluorescence intensity
RGD: Tripeptide arginine-glycine-aspartic acid
SMA: Smooth muscle actin
SolEng: Soluble endoglin
TGF-β: Transforming growth factor β
UASMC: Umbilical artery smooth muscle cells
VSMC: Vascular smooth muscle cells
ZP: Zona pellucida
