# Supplementary material for: Endoglin regulates mural cell adhesion in the circulatory system
Source: Cell Mol Life Sci. 2015 Dec 8;73(8):1715–39. doi: 10.1007/s00018-015-2099-4 (PMC4805714; doi:10.1007/s00018-015-2099-4)
Supplement: Supplementary file 1 — Supplementary material 1 (PDF 15639 kb) [file 18_2015_2099_MOESM1_ESM.pdf]

SUPPLEMENTAL MATERIAL

ENDOGLIN REGULATES MURAL CELL ADHESION IN THE CIRCULATORY SYSTEM

Elisa Rossi[1,2,3], David M. Smadja[3,4], Elisa Boscolo[5], Carmen Langa[1], Miguel A. Arevalo[6,7], Miguel Pericacho[7,8], Luis Gamella-Pozuelo[1,8], Alexandre Kauskot[9,10], Luisa M. Botella[1], Pascale Gaussem[3,4], Joyce Bischoff[5], José M. Lopez-Novoa[7,8], Carmelo Bernabeu[1*]

[1]Centro de Investigaciones Biológicas, Consejo Superior de Investigaciones Científicas (CSIC), and Centro de Investigación Biomédica en Red de Enfermedades Raras (CIBERER), 28040 Madrid, Spain
[2]Paris Descartes University, Sorbonne Paris Cite, Paris, France
[3]AP-HP, Hôpital Européen Georges Pompidou, Hematology Department, Paris, France
[4]Inserm UMR-S1140, Faculté de Pharmacie, Paris, France
[5]Department of Surgery, Harvard Medical School, Children's Hospital, Boston, MA 02115, USA
[6]Departamento de Anatomía e Histología Humanas, Facultad de Medicina, Universidad de Salamanca, 37007 Salamanca, Spain
[7]Instituto de Investigaciones Biomédicas de Salamanca (IBSAL), 37007 Salamanca, Spain
[8]Departamento de Fisiología y Farmacología, Unidad de Fisiopatología Renal y Cardiovascular, Universidad de Salamanca, 37007 Salamanca, Spain
[9]Inserm UMR-S1176, Le Kremlin Bicêtre, France
[10]Université Paris Sud, Le Kremlin Bicêtre, France

Expanded Materials and Methods

Cell lines and primary cultures

The Jurkat (T cell lymphoblast-like) cell line was cultured in RPMI 1640 supplemented with 10% heat inactivated fetal calf serum (FCS), 2 mM L-glutamine, 100 U/mL penicillin and 25 µg/mL gentamycin. Human umbilical vein endothelial cells (HUVECs), and human aortic endothelial cells (HAECs) (Lonza, Walkersville, USA) were grown on gelatin (Sigma) coated wells using EBM-2 medium, supplemented with EGM-2 SingleQuots (Lonza) and 10% FCS. Umbilical artery smooth muscle cells (UASMCs; Lonza) were grown in SmGM2 bullet kit medium (Lonza), supplemented with 10% FCS. Experiments with these primary cells were carried out at passages 4-8. Among endothelial progenitor cells (EPCs), endothelial colony-forming cells (ECFCs) exhibit features of a true endothelial progenitor population [1,2]. ECFCs were isolated as described [3] and were grown on gelatin coated wells using EBM-2 medium, supplemented by 20% FCS and by EGM-2 SingleQuots (Lonza). ECFCs are able to form the endothelial lining of blood vessels when subcutaneously injected in combination with smooth muscle cells or bone marrow-derived mesenchymal progenitor cells (bmMPCs) in mice [4,5] bmMPCs were grown on fibronectin (Millipore) coated wells using EBM-2 medium, supplemented by 20% FCS and by rhFGF-B, R3-IGF-1, ascorbic acid, and GA-1000 (Lonza). bmMPCs were differentiated into pericytes by seeding bmMPCs together with ECFCs at a ratio 1:1 at a total density of 10^4cells/cm^2 on fibronectin-coated plates in EBM-2 medium containing 20% FCS. All cell types were cultured in a 5% CO_2 atmosphere at 37ºC. For *in vitro* wound healing assays, cells were grown to confluence and multiple wounds were created by scratching the cell monolayer using a sterile pipette tip. Plates were immediately washed with PBS to remove the remaining cells and fresh medium was added. Cells were photographed and collected at the times indicated, and analyzed for endoglin expression by immunofluorescence flow cytometry.

Expression and silencing vectors and cell transfections

The expression vector encoding HA-tagged full length endoglin (Endo-FL; amino acids 26-658) and the derived truncated construct 437/586-Endo (amino acids 437-586) in pDisplay (Invitrogen) have been previously reported [6]. The pCEXV-EndoL plasmid, encoding human L-endoglin [7], was used to derive by PCR amplification a truncated endoglin construct containing the Zona Pellucida domain (ZPD; amino acids 340-658). The oligonucleotides used to prime the PCR synthesis of the endoglin fragment were as follows: ZPD-Endo (amino acids 340–658), 5´-GCATAGATCTCCGATCCAGACCACTC-3´ (forward) and 5´-ATACCGCGGCTATGCCATGC-3´ (reverse). The oligonucleotides were designed to introduce a *BglII* restriction site at the 5´-end and a *SacII* site at the 3´-end and the PCR fragment was inserted into the *BglII/SacII* sites of pDisplay. The resulting ZPD-Endo-pDisplay vector was used to generate a mutant of the RGD motif where the Asp401 residue is replaced by Ala. Site directed mutagenesis was performed using 5´-GGACAGGGGTGCCAAGTTTGTCT-3´ (forward) and 5´-AGACAAACTTGGCACCCCTGTCC-3´ (reverse) leading to the ZPD-Endo-RGA-pDisplay vector. All endoglin constructs expressed from

pDisplay contain the influenza hemagglutinin epitope HA at the NH_2 terminus. To silence endoglin expression, siRNAs (s4677 and s4679) and scrambled siRNAs (AM4611 and AM4613), as negative controls (Ambion, Life Technology), were used. Nucleofections with expression and silencing vectors were performed following the manufacturer's instructions with Amaxa nucleofector kits VCA-1003 (Jurkat cell line), VPB-1002 (HUVECs), and VPI-1004 (UASMCs) from Lonza using the Nucleofector I (AMAXA, Germany). Silencing experiments were carried out by simultaneously nucleofecting two endoglin siRNAs (s4677 and s4679) using scrambled siRNAs #1 (AM4611) and #2 (AM4613), as negative controls. Alternatively, silencing of endoglin was performed using endoglin siRNA (sc-35302; Santa Cruz Biotech) and scrambled siRNA (AllStars; Qiagen #1027280), as a negative control, in the presence of PrimeFect siRNA transfection reagent and PrimeFect diluent (PA3269 and PA3271; Lonza). Silencing of β1 integrin was performed using β1 integrin siRNA (sc-35674; Santa Cruz Biotech) and scrambled siRNA (AllStars; Qiagen #1027280), as a negative control, in the presence of PrimeFect siRNA transfection reagent and PrimeFect diluent (PA3269 and PA3271; Lonza). To monitor the suppression efficiency, immunofluorescence flow cytometry to detect endoglin or β1 integrin expression was performed on all cell types. Samples were also analyzed by confocal microscopy (Confocal Laser Scanning Microscope, CLSM, Leica TCS SP5).

Antibodies and other reagents

The following antibodies were used: Rabbit polyclonal anti-human endoglin (clone H-300, sc-20632; Santa Cruz Biotechnology), mouse monoclonal antibody (mAb) P4A4 anti-human endoglin (IgG2b; Developmental Studies Hybridoma Bank, University of Iowa, Iowa City, IA), mouse anti-human von Willebrand Factor mAb (clone F8/86, M0616, Dako), normal rabbit IgG (sc2027, Santa Cruz Biotechnology), normal mouse IgG (sc2025, Santa Cruz Biotechnology), rabbit anti-calponin antibody (EP798Y, ab46794, Abcam), goat anti-Sm22α (ab10135 Abcam), rabbit anti-PDGFRβ (P-20, sc-339, Santa Cruz Biotechnology), mouse anti-human NG2/MCSP IgG1 mAb (MAB2585, R&D System), anti-αsmooth muscle actin (αSMA) mAb (clone 1A4, A5528, Sigma-Aldrich), anti-podocin rabbit mAb (anti-NPHS2, EPR13820, ab181143 Abcam), anti-Willms Tumor (WT1) (Santa Cruz Biotechnology c-19, sc-192), anti-CD31 (1A10, Leica Biosystems), anti-CD68 (Dako Diagnostics), anti-X63 mAb (with no known cross reactivity; negative control), IgG2b mAb (Immunostep, Salamanca, Spain), TS2/16 (anti-β1) and LIA1/2 (anti-β1, inhibitory) mAbs kindly provided by Dr. Francisco Sanchez-Madrid (Hospital de la Princesa, Madrid, Spain), anti-CD29 (β1 integrin) mouse mAb (clone MEM-101A; Invitrogen, Life Technologies), anti-CD105 mouse mAb (1:100 dilution; DAKO Diagnósticos, Barcelona, Spain), mouse anti-CD184 (CXCR4) (1:100 dilution; BioLegend, San Diego, CA), Alexa Fluor® 488 conjugate (MHCD10520, Invitrogen), anti-hemagglutinin (HA) mAb (Roche Diagnostics), anti-human IgG (Fc specific)-FITC (F9512 Sigma-Aldrich), Fluorescein (FITC) anti-goat IgG (FI-5000, Vector), FITC anti-rabbit IgG (FI-1000, Vector), FITC anti-mouse IgG (FI-2000, Vector), Texas Red anti-mouse IgG (TI-2000 Vector), Texas Red anti-rabbit IgG (TI-1000 Vector), Alexa-488 anti-mouse IgG, Alexa 568 anti-mouse IgG, anti-rabbit IgG, and Alexa-647 anti-mouse IgG (Invitrogen). All the

antibodies were used strictly following their datasheets. Human SolEng and CXCL12 were from R&D Systems (MN, USA). The tripeptide RGD (Arg-Gly-Asp) was from Sigma-Aldrich and the pentapeptides GRGDS (RGD), SDGRG (DGR; used as a negative control), and the human endoglin-derived DRGDK (RGDK) were synthesized in the Centro de Investigaciones Biologicas (Madrid, Spain), using a peptide synthesizer (AAPPtec, Focus XC) with Fmoc chemistry. These peptides were used at 0.5 or 1mM in cell assays.

Binding assays of SolEng to cells

The chimeric construct EndoEC-Fc, encoding the extracellular domain of endoglin fused to the Fc fragment of IgG has been described [8]. UASMCs were cultured in 6-well plates (Falcon, Becton Dickinson Labware Europe). Exponentially growing cells were incubated in complete EBM-2 medium either in the presence or in the absence of an integrins activator (CXCL12 at 200ng/mL or $MnCl_2$ at 200μM) for 20 min at 37ºC. Cells were washed with PBS and then incubated with different combinations of EndoEC-Fc, RGD peptide (1mM) or DGR peptide (1mM) in 2% AB^+ human serum in PBS for 10 min at 37ºC. Samples were washed with PBS and FITC-labeled secondary antibody anti-human IgG (Fc specific) was added for 1h at 4ºC. Samples were washed with PBS and fixed with 10% formaldehyde. Next, the Vectashield mounting medium for fluorescence with DAPI (H-1200, Vector) was used to counterstain the samples and analyze them by confocal microscopy (Confocal Laser Scanning Microscope, CLSM, Leica TCS SP5) and video recording (Leica Las AF Lite).

Immunofluorescence flow cytometry

Adherent cells (HUVEC, HAEC, UASMC) after trypsin treatment or cell lines in suspension (Jurkat) were incubated for 30 minutes at 4ºC with 2% human AB^+ serum in PBS (blocking solution) and then for 1 hour at 4ºC with the primary antibody P4A4 specific for human endoglin (DSHB, University of Iowa, USA; Dr. Elizabeth A. Wayner), the anti-CD29 (β1 integrin) mouse mAb (clone MEM-101A; Invitrogen, Life Technologies) or with the mAb X63 as a negative control. After two washes with cold PBS, samples were incubated with the secondary antibody (Alexa 488 anti-mouse, Invitrogen). Finally, cells were washed twice, and their fluorescence was measured in a Coulter Epics XL flow cytometer (Beckman Coulter, High Wycombe, UK). When necessary, cells nucleofected with GFP were also analyzed by fluorescence flow cytometry. For double fluorescence analysis, cells were selected on basis of forward and side scatter characteristics and the results were represented in a dot plot showing events (cells) for the two fluorescence signals (675 nm for Alexa 647 and 525 nm for GFP), using an FC-500 flow cytometer (Beckman Coulter).

Immunofluorescence of 2D co-culture

Culture chamber slides (Millicell EZ slide, 8 well glass, Millipore #PEZGS0816, Millipore Ireland Ltd., Cork, Ireland) were coated with fibronectin and seeded with ECFC and bmMPC at a 1:1 ratio. After *in vitro* co-culture for seven days, bmMPC differentiate into VSMC/pericytes, as evidenced by the positivity with antibodies specific for αSMA, PDGFRβ, NG2, calponinI and Sm22α [4]. Once differentiated, cells were fixed with cold pure methanol on ice for 10 min. and washed twice with PBS.

For immunostaining of ECFC, samples were incubated with a mAb anti-human von Willebrand factor (Dako, dilution 1:500) for 1h at room temperature, followed by washing 3 times with PBS, incubation with the secondary antibody Texas Red anti-mouse IgG (TI-2000 Vector, dilution 1:200) for 1h at room temperature, and washing 3 times with PBS. For staining of differentiated bmMPC, the same samples were incubated with anti-human calponin (EP798 #ab46794 Abcam, dilution 1:100), anti-human Sm22α (#ab10135, Abcam, dilution 1:100), anti-PDGFRb (sc-339, Santa Cruz, dilution 1:100), anti-human NG2 (R&D Systems, dilution 1:500), anti-human αSMA (Sigma, dilution 1:500) or a negative control antibody (normal rabbit IgG #sc2027, normal goat IgG #sc2028, or normal mouse IgG #sc2025, dilution 1:100; Santa Cruz). After 3 washes with PBS, samples were incubated with the appropriate secondary antibody diluted 1:200: FITC anti-goat IgG (#FI-5000,Vector), FITC anti-rabbit IgG (#FI-1000, Vector) or FITC anti-mouse IgG (#FI-2000, vector). After washing, samples were mounted using Vectashield with DAPI (#H1200 Vector). In the final samples, endothelial cells (ECFC) are stained in red (anti-von Willebrand factor), pericytes are stained in green (pericyte markers) and nuclei are stained in blue (DAPI).

Cell adhesion assays

Cultures of UASMCs in 24-well plates (Falcon, Becton Dickinson, Oxnard, CA; NuncTM Brand Products, Denmark) were used as a cell substrate in adhesion assays with the Jurkat cell line. When UASMCs were 90% confluent, Jurkat cells nucleofected with human endoglin and GFP constructs were added to the wells. After 1 hour incubation, wells were rinsed twice with PBS and photographed by confocal microscopy. Then, bound cells were lysed with TNE 10x (0.5M Tris, 1.5M NaCl, 10mM EDTA; pH 7.5) and their fluorescence was measured. When necessary, UASMCs were stained with a red vital stain (CellTracker™ Red CMTPX, Molecular Probes, Invitrogen, Life Technologies) for 30 min at 37ºC, and then washed with PBS prior to the cell adhesion assay. The quantification of substrate adherence capacity was carried out by a fluorescent analyser (Varioskan Thermo-Fisher Scientific), selecting the appropriate wavelength (GFP: 494-515nm). A similar method was used to quantify the adhesion of pericytes to endothelial cells. Briefly, HUVEC monolayers or pericyte monolayers stained with CMTPX were incubated with pericytes or HUVECs, respectively, previously labeled with the green stain CSFE (CellTracker™, Molecular Probes, Invitrogen, Life Technologies) in the presence or in the absence of SolEng (50ng/mL; R&D Systems), CXCL12 (100ng/mL; R&D Systems), RGD, RGDK, or DGR peptides (1mM), or $MnCl_2$ (200ng/mL). After 1 hour of incubation, wells were subjected to lysis and adhesion of pericytes to HUVECs was measured using the Varioskan equipment selecting the appropriate wavelength (CSFE: 494-515nm) as above. Images of adhesion between cells in suspension and adherent cells or between HUVECs and pericytes were obtained by confocal microscopy (Confocal Laser Scanning Microscope, CLSM, Leica TCS SP5). Quantification of bound cells was carried out by measuring the surface area of the cells using ImageJ Version 1.46 software. When required, UASMCs or HAECs were preincubated with 100ng/mL CXCL12 for 30 min at 37ºC,

prior to CSFE labeling and cell adhesion assays. The concentrations of cycloheximide and monensin were selected from an initial dose-dependent study to exclude possible toxic effects of the drugs.

Phosphorylation and Western blot studies

Individual and combined cultures of UASMCs and HAECs were incubated in the absence or presence of 1µg/mL SolEng. At different time points, adherent cells were lysed in modified RIPA buffer (1% Triton X-100, 1% sodium deoxycholate, 0.1% SDS, 50 mM HEPES, 10% glycerol, 150 mM NaCl, 1.5 mM $MgCl_2$, 1 mM EGTA, 1 mM $NaVO_3$, 100 mM $Na_4P_2O_7$, 100 mM NaF, 10 µg/ml aprotinin and 5 µg/ml leupeptin, pH 7.4). The lysates were then clarified by centrifugation at 15,000 g for 10 minutes at 4ºC. Proteins were subjected to SDS-PAGE and transferred to nitrocellulose. The membranes were incubated with the primary antibodies anti-p-FAK (Tyr925; BD Biosciences, 1/1000), anti-p-Akt (Ser473; Cell Signalling, 1/500) and anti-actin (Sigma, 1/10,000). Immunoreactive bands were visualized using enhanced chemiluminescence detection reagents (Pierce). Images of chemiluminescent signals were captured using G:BOX Chemi XT16 Image Systems and quantified using Gene Tools version 4.0.0.0 (Syngene). The presence of HA-tagged SolEng in concentrated urine samples was detected by Western blot analysis with anti-endoglin (P4A4) and anti-HA antibodies, using X63 as a negative control. Endoglin protein bands were visualized with a ChemiDoc™ XRS+ equipment (Bio-Rad, Madrid, Spain) and their intensity was quantified using Image Lab™ software.

3D coculture of vascular ECs and mural cells

3D cocultures of endothelial cells and UASMCs at a 4:1 ratio, respectively, were performed using Matrigel (BD Bioscience). When necessary, adherent endothelial cells (HUVEC/HAEC; 80,000 cells/well) stained in red (CMTPX) and smooth muscle cells (UASMC) stained in green (CSFE) were co-cultured. Similarly, HUVECs, HAECs, UASMCs and adherent cells nucleofected with siRNA/GFP were resuspended and cocultured in Matrigel. Endothelial cells (HUVECs/HAECs/ECFCs) and mural cells (UASMC, bmMPC) were resuspended in a mixture of Matrigel:Medium (1:1) in a total volume of 100µL/well. For *in vitro* assays, 4-well glass slides with a 1.7 cm^2 growing area per well (Millipore) were used. Additives were added at time 0 and cocultures were monitored for 6h at 37ºC. After 4h of incubation, photographs of the slides were taken. For studying the potential role of integrins we used the mAbs TS2/16 (anti-β1, integrin activating at 20 µg/mL) and LIA1/2 (anti-β1, integrins inhibiting 10µg/mL and 20 µg/mL) and an IgG2b mAb (Immunostep, Salamanca, Spain), which reacts with an undefined epitope on a plant pathogen, was used as a negative control. As a global integrin activator $MnCl_2$ at 200µM was used. For checking the potential involvement of CXCL12 and CXCR4, we used the macrocyclic compound AMD3100 octahydrochloride (Sigma-Aldrich Inc., Spain), inhibitor of the CXCR4 alpha-chemokine receptor and its ligand CXCL12, at a concentration of 5 µM. Human SolEng (1µg/mL), RGD (Arg-Gly-Asp; 1mM), PDGF-BB (10ng/mL) and CXCL12 (200ng/mL) were also used.

Immunohistochemistry and ELISA

Sections (3µm) from kidneys, lungs and Matrigel plugs were cut and stained with hematoxylin and eosin or with Masson's trichrome stain, as indicated. For immunohistochemical analysis, sections were deparaffinated in xylene and rehydrated in a graded series of ethanol. The antigen-retrieval process was carried out by a 3-min microwave incubation of sections with citrate solution (BioGenex, CA). Endogenous peroxidase was blocked by incubation in 3% hydrogen peroxide and sections were incubated with anti-αSMA (ASM-1 at 1:2,000 dilution; Leica Biosystems), anti-podocin (ab181143 at 1:700 dilution; Abcam), anti-Willms Tumor (WT1) (sc-192 at 1:800 dilution; Santa Cruz Biotechnology), anti-CD31 (1A10 at 1:100 dilution, Leica Biosystems), mouse monoclonal anti-CD105 (1:100 dilution; DAKO Diagnósticos, Barcelona, Spain) or anti-CD68 (1:100 dilution; Dako Diagnostics, Copenhagen, Denmark). Then, sections were washed three times in PBS and incubated with the Novolink Polymer Detection System (Novocastra, MA, USA), followed by reaction with 3,3´diaminobenzidine as chromogen. Negative controls were performed in the absence of the primary antibody. For ThinPrep cytology, urine cytological slides were prepared by using a liquid-based method technique following the manufacturer's guidelines (ThinPrep 2000; Cytyc, Marlborough, MA) and stained using either the standard hematoxylin technique or immunohistochemistry against αSMA as above. Urine samples were used to determine the concentration of SolEng (R&D Systems, Minneapolis, MN), nephrin (Exocell, Philadelphia, PA) and podocalyxin (Wuhan EIAAB Science, Wuhan, China), using commercial ELISA kits.

Mice and in vivo assays

For Matrigel plug assays, ECFCs and bmMPCs (1:1 ratio; total cells $3x10^6$) were resuspended in 200 µL of Matrigel (BD Bioscience, Le Pont de Clais, France) either in the absence or in the presence of human SolEng and subcutaneously injected into 6-week-old male nude mice (n=13). In addition, similar Matrigel plug assays were carried out in the absence of cells. After 7 days, intact Matrigel plugs were carefully exposed, fixed, and embedded in paraffin for histological examination. Three different sections of each plug were analyzed and blood vessels were quantified by hematoxylin and eosin staining. Transgenic C57BL/6J mice (12-20 weeks of age) with recurrent backcrossing, expressing an HA-tagged soluble form of endoglin, driven by a ubiquitous actin promoter (*Sol.Eng*$^+$) [9], were used for immunohistochemistry of the kidney and lung. Representative tissue stainings of *Sol.Eng*$^+$ (n=17) and WT (n=17) mice are shown in the figures. Urine from *Sol.Eng*$^+$ (n=10) and WT (n=10) mice was collected in metabolic cages or obtained directly from the bladder. Urinary cytology was performed using the ThinPrep technique. The concentration of endoglin and the podocyte markers nephrin and podocalyxin was measured by ELISA and urinary cells were stained with podocyte-specific antibodies. Vascular permeability studies were carried out in *Eng*$^{+/-}$ mice and their *Eng*$^{+/+}$ littermates [10,11]. Mice were anesthetized with 1.2% isoflurane and perfused through the jugular vein with a solution of fluorescein isothiocyanate-dextran (FITC-dextran; $2x10^6$ kDa, F2000S; Sigma Aldrich, St. Louis, MO) at 100 mg/kg body weight. After two hours, mice were sacrificed by cervical dislocation and eyes were

removed, fixed in 4% paraformaldehyde for 1 hour and incubated for 1 hour in PBS. Eyes were dissected and the neuroretinas (n=19) were isolated and mounted with antifade solution (ProLong gold antifade reagent, Life Technologies). Retinas were photographed using a fluorescence microscope (Axiovert 200M, Zeiss). All procedures were approved by the Animal Care and Use Committees of the University of Salamanca and University of Paris. Also, mice were cared for in accordance with the standards established in the National Institutes of Health Guide for the Care and Use of Laboratory Animals.

Cell quantification and statistical analysis

Binding of UASMCs to HUVECs in angiogenesis assays was quantified by measuring the intensity profile using fluorescence confocal microscopy (SP5, Leica). Fluorescence images were transformed in binary system by ImageJ to quantify the percentage of cell adhesion. In adhesion assays, bound fluorescently labeled cells were lysed and quantification was carried out using a Varioskan plate reader. All values are expressed as mean ± standard deviation. Results are representative of, at least, 5 independent experiments in triplicate samples. Statistical analysis was performed using a one-way ANOVA and Bonferroni post-test. A value of $p<0.05$ (*) was considered statistically significant and a value of $p<0.005$ (**) or $p<0.001$ (***) was considered statistically highly significant.

References

1. Smadja DM, Bièche I, Uzan G, Bompais H, Muller L, Boisson-Vidal C, Vidaud M, Aiach M, Gaussem P (2005) Par-1 activation on human late endothelial progenitor cells enhances angiogenesis in vitro with upregulation of the sdf-1/cxcr4 system. Arterioscler Thromb Vasc Biol 25:2321-2327.
2. Smadja DM, Bièche I, Silvestre JS, Germain S, Cornet A, Laurendeau I, Duong-Van-Huyen JP, Emmerich J, Vidaud M, Aiach M, Gaussem P (2008) Bone morphogenetic proteins 2 and 4 are selectively expressed by late outgrowth endothelial progenitor cells and promote neoangiogenesis. Arterioscler Thromb Vasc Biol 28:2137-2143.
3. Khan ZA, Melero-Martin JM, Wu X, Paruchuri S, Boscolo E, Mulliken JB, Bischoff J (2006) Endothelial progenitor cells from infantile hemangioma and umbilical cord blood display unique cellular responses to endostatin. Blood 108:915-921.
4. Boscolo E, Stewart CL, Greenberger S, Wu JK, Durham JT, Herman IM, Mulliken JB, Kitajewski J, Bischoff J (2011) Jagged1 signaling regulates hemangioma stem cell-to-pericyte/vascular smooth muscle cell differentiation. Arterioscler Thromb Vasc Biol 31:2181-2192.
5. Melero-Martin JM, De Obaldia ME, Kang SY, Khan ZA, Yuan L, Oettgen P, Bischoff J (2008) Engineering robust and functional vascular networks in vivo with human adult and cord blood-derived progenitor cells. Circ Res 103:194-202.

6. Guerrero-Esteo M, Sanchez-Elsner T, Letamendia A, Bernabeu C (2002) Extracellular and cytoplasmic domains of endoglin interact with the transforming growth factor-beta receptors I and II. J Biol Chem 277:29197-29209.
7. Bellon T, Corbí A, Lastres P, Calés C, Cebrián M, Vera S, Cheifetz S, Massague J, Letarte M, Bernabéu C (1993) Identification and expression of two forms of the human transforming growth factor-beta-binding protein endoglin with distinct cytoplasmic regions. Eur J Immunol 23:2340-2345.
8. Blanco, Santibanez JF, Guerrero-Esteo M, Langa C, Vary CP, Bernabeu C (2005) Interaction and functional interplay between endoglin and alk-1, two components of the endothelial transforming growth factor-beta receptor complex. J Cell Physiol 204:574-584.
9. Valbuena-Diez AC, Blanco FJ, Oujo B, Langa C, Gonzalez-Nuñez M, Llano E, Pendas AM, Díaz M, Castrillo A, Lopez-Novoa JM, Bernabeu C (2012) Oxysterol-induced soluble endoglin release and its involvement in hypertension. Circulation 126:2612-2624.
10. Bourdeau A, Dumont DJ, Letarte M (1999) A murine model of hereditary hemorrhagic telangiectasia. J Clin Invest 104:1343-1351.
11. Jerkic M, Rivas-Elena JV, Santibanez JF, Prieto M, Rodríguez-Barbero A, Perez-Barriocanal F, Pericacho M, Arévalo M, Vary CP, Letarte M, Bernabeu C, López-Novoa JM (2006) Endoglin regulates cyclooxygenase-2 expression and activity. Circ Res 99:248-256.

SUPPLEMENTARY FIGURE LEGENDS

Supplemental Figure 1. Effect of in vitro wound healing on endoglin expression. HAECs, UASMCs and bmMPCs were grown to confluence and multiple wounds were created by scratching the cell monolayer using a sterile pipette tip. Plates were washed with PBS to remove the remaining cells and fresh medium was added. At different times after wounding, cells were photographed (**A**) and collected for immunofluorescence flow cytometry using anti-endoglin antibodies (**B,C**), and analyzed for endoglin expression. In **C**, a comparison of the mean fluorescence intensities between wounded and unwounded cells is shown.

Supplemental Figure 2. Effect of soluble endoglin and CXCL12 on UASMC adhesion to HAECs. **A.** UASMCs, previously labeled with CSFE (green) were cocultured with unlabeled HAECs at a 1:4 ratio in matrigel to analyze mural cell adhesion to ECs. Cells were untreated (Control) or treated in the presence of soluble endoglin (1µg/mL) or CXCL12 (0.2µg/mL), as indicated. The left column shows representative photographs of confocal microscopy analysis. The right column represents the intensity profile of SMC adhesion measured using fluorescence confocal microscopy (SP5, Leica) using a 3D reconstruction. **B,C.** UASMCs express the CXCL12 specific receptor CXCR4. UASMCs were

incubated with anti-CXCR4 or X63 hybridoma supernatant (IgG), as a negative control. Samples were analyzed by immunofluorescence flow cytometry (**B**) and confocal microscopy (**C**).

Supplemental Figure 3. Silencing of endoglin in HAECs. Primary cultures of HAECs were transfected with endoglin specific siRNA (siRNA-Eng) or scrambled siRNA (scRNA). Transfected HAECs were morphologically (**A**), phenotypically (**B**) and functionally (**C,D**) analyzed. **A**. Untreated cells and cells transfected with endoglin specific siRNA or scrambled siRNA display a similar morphology. **B**. Immunofluorescence flow cytometry with anti-endoglin antibodies show a downregulation (89%) of endoglin in cells transfected with specific siRNA *versus* cells transfected with scrambled siRNA. **C**. Cell-cell adhesion assays. Confluent monolayers of UASMCs, transfected with endoglin specific siRNA or scrambled siRNA, were incubated with HAECs previously labeled with CFSE, in the absence (control) or presence of 1μg/mL soluble endoglin (Sol.Eng) or 100ng/mL CXCL12, as indicated. After one hour incubation, wells were washed and the cells were visualized by confocal microscopy. **D**. Binding of UASMCs to HAECs in panel **C** was quantified by measuring the fluorescence intensity using Image J and Histolab™ (Microvision) software. (**p<0.005; ***p<0.001).

Supplemental Figure 4. Expression of mutant endoglin constructs upon transfection. **A.** Western blot analysis. Human embryonic kidney cells HEK293T were transfected, using Lipofectamine LTX and Plus reagent (Invitrogen), with endoglin expression vectors, as indicated. After 48 hours, total lysates were subjected to SDS-PAGE under reducing conditions, followed by Western blot with anti-HA antibody. The specific endoglin bands are indicated by arrows. The asterisk indicates the presence of a non-specific band recognized by the anti-HA antibody. The molecular weight (Mr) values in kDa are shown. Endoglin-FL, endoglin full length; Control, empty vector. The nomenclature for endoglin mutants is as in Figure 5. **B.** Flow cytometry analysis. Jurkat cells were nucleofected with full length (FL) endoglin, ZPD-Endo, ZPD-Endo-RGA or 437/586-Endo constructs together with GFP. As negative controls, cells were nucleofected with GFP in the absence (Control) or in the presence of the empty vector. Two days after nucleofection, cells were collected and analyzed by immunofluorescence flow cytometry with anti-HA antibodies (red Alexa 647) and GFP (green) using a Cytomics FC500 equipment (Beckman Coulter). Anti-HA antibodies recognized all endoglin constructs at the cell surface. Co-transfection efficiency was calculated by measuring the number of cells that simultaneously express endoglin constructs at the cell surface as well as GFP. Co-transfection efficiency (HA and GFP) respect to the total number of cells is indicated. In the case of cells transfected with empty vector, the single efficiency of GFP transfection is indicated in parenthesis.

Supplemental Figure 5. Effect of soluble endoglin on *in vivo* angiogenesis. A. Characterization of ECFCs. Immunofluorescence flow cytometry for endoglin (CD105) on ECFCs and HUVECs using a flow cytometer (top panels). The fluorescence intensity is indicated in the vertical axis, whereas the forward scatter values (FSC-H) are represented in the horizontal axis. The number in the upper right corner represents the percentage of endoglin positive cells. Analysis by contrast microscopy shows that

ECFCs exhibit the typical cobblestone morphology as HUVECs (bottom panels). **B.** Sections of Matrigel plugs containing bmMPCs and ECFCs (with or without soluble endoglin) from the experiment in Figure 6 were analyzed by immunohistochemistry. Plug sections were stained with hematoxylin and eosin (H&E). Arrowheads indicate the presence of vascular structures containing erythrocytes (H&E staining) or mural cells stained with an antibody to αSMA. Staining of αSMA is almost absent in plugs treated with soluble endoglin. The number of vessels was higher in control plugs versus those treated with soluble endoglin.

Supplemental Figure 6. Immunohistochemistry of kidney glomeruli and analysis of urine. A,B. Analysis of podocytes in the kidney glomeruli from *Sol.Eng*$^+$ (n=17) and control (n=17) mice. Tissue sections were stained with anti-podocin (**A**) or anti-αSMA (**B**) antibodies. Podocytes can be identified by podocin immunostaining (see arrows in **A**). Overall, immunostaining with anti-αSMA and anti-podocin shows a positive signal in podocytes from wild type animals and a reduced stain in *Sol.Eng*$^+$ glomeruli. A quantification of the mural cell immunostaining is indicated in Figure 9B. Some examples with a markedly reduced signal of αSMA and podocin in *Sol.Eng*+ glomeruli compared to control animals are included. **C.** Detection of endoglin in glomeruli from *Sol.Eng*$^+$ and wild type mice. Representative tissue sections stained with anti-endoglin antibodies. The staining of endoglin is higher (arrows) in glomeruli of *Sol.Eng*$^+$ mice compared to control mice. **D.** Detection of soluble endoglin in the urine of *Sol.Eng*$^+$ mice. Urine samples from *Sol.Eng*$^+$ (#1 and #2) and control (#1 and #2) mice were concentrated and subjected to SDS-PAGE under non reducing conditions, followed by Western blot with anti-endoglin antibodies. The arrow indicates the presence of a 140-kDa band corresponding to the soluble endoglin dimer present in *Sol.Eng*$^+$ mice, but not in mice. Φ indicates the presence of unspecific bands. The asterisk indicates the presence of high molecular weight oligomers of endoglin. The molecular weight (Mr) values in kDa are shown. A representative experiment out of three different ones is shown.

Supplemental Video #1. Three dimensional reconstruction of soluble endoglin bound to VSMCs. Exponentially growing UASMCs were incubated with 2% AB$^+$ human serum in PBS for 20 min at 37ºC in the presence of a chimeric protein containing the extracellular domain of endoglin fused to the Fc fragment of IgG (Sol.Eng-Fc), and the integrins´ activator CXCL12. Samples were washed with PBS and FITC-labeled secondary antibody anti-human IgG (Fc specific) was added for 1h at 4ºC. Cells were fixed with 10% formaldehyde and mounted with Vectashield medium combined with DAPI counterstaining. Samples were analyzed using fluorescence confocal microscopy (SP2, Leica) as shown in Fig. 5A. The representative video shows a 3D reconstruction made using LAS AF Lite software (Leica). The endoglin fluorescence is located at cell surface.

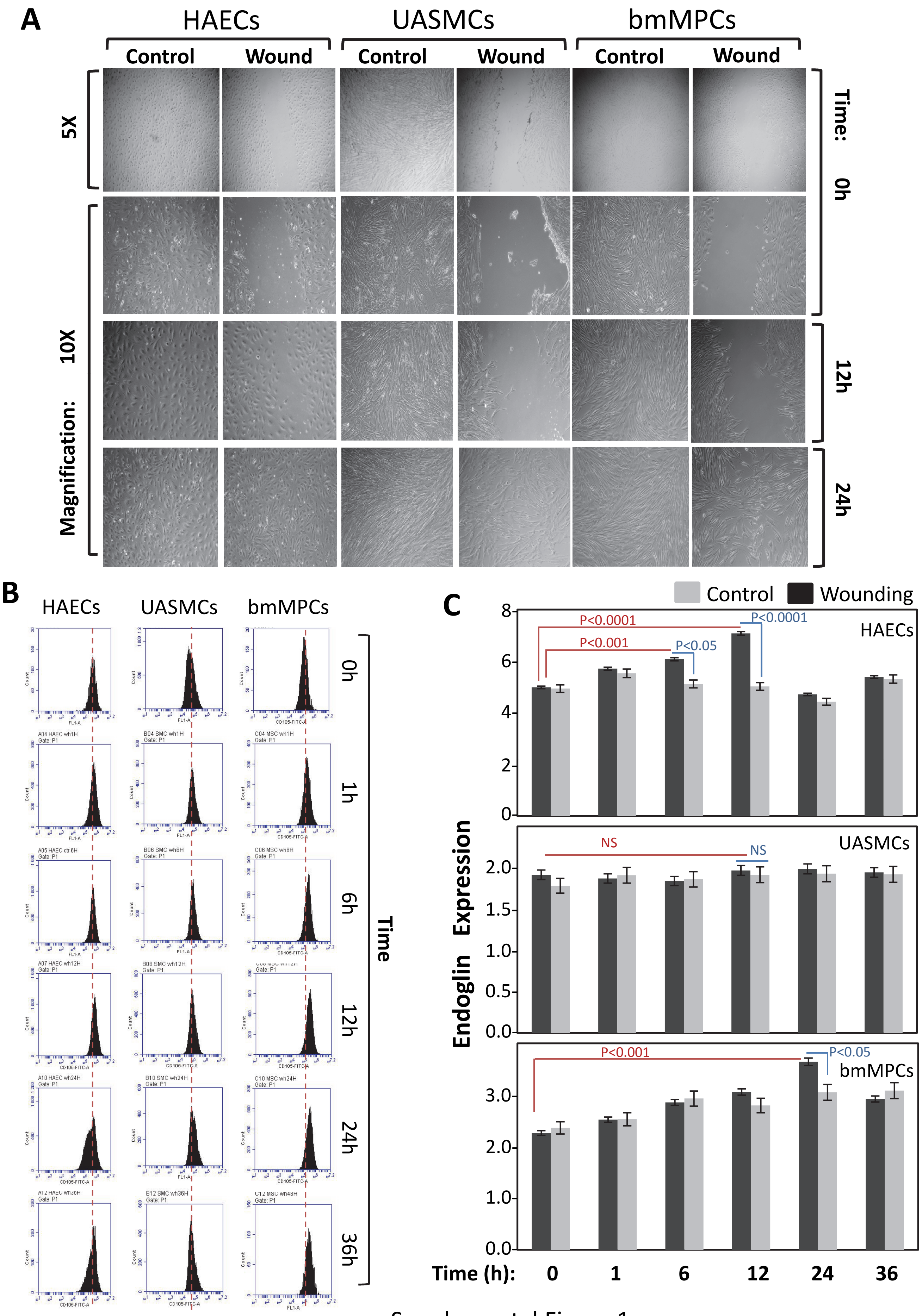

Supplemental Figure 1

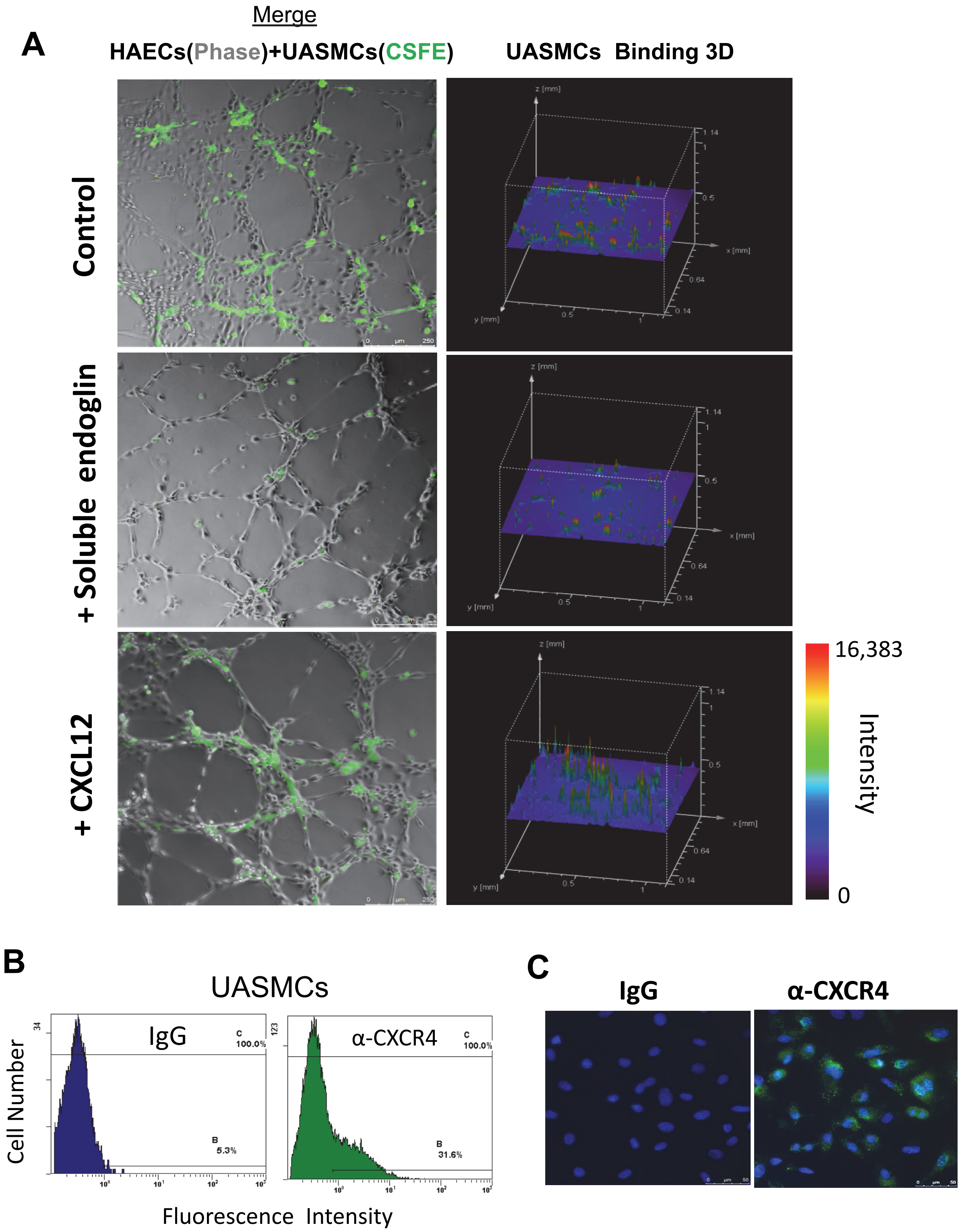

Supplemental Figure 2

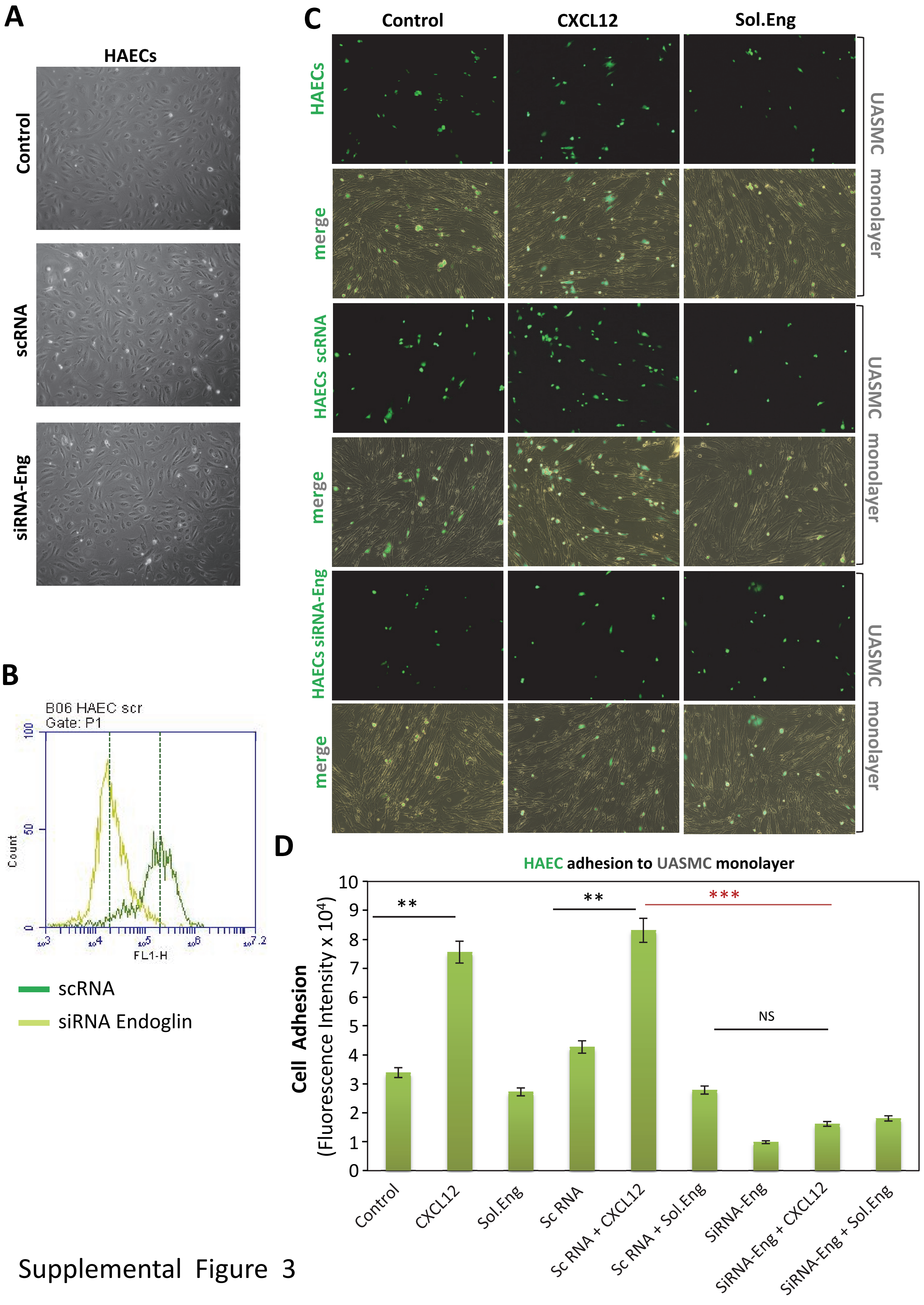

Supplemental Figure 3

A

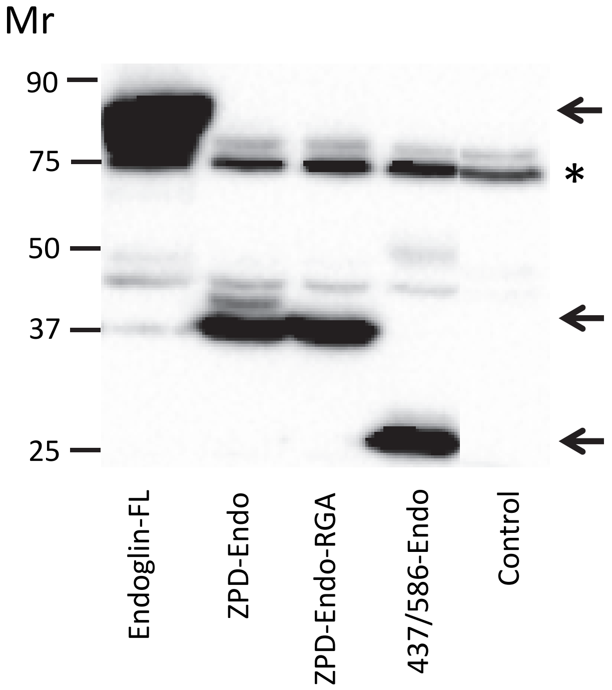

B

**Conditions**	**Co-transfection efficiency**
Control	0.70% ± 0.35
pDisplay-Empty vector	0.90% ± 0.10 (GFP:46.5%)
pDisplay-Endoglin-FL	29.25% ± 4.75
pDisplay-ZPD-Endo	35.35% ± 4.65
pDisplay-ZPD-Endo-RGA	25.50% ± 5.50
pDisplay-437/586-Endo	26.75% ± 3.25

Supplemental Figure 4

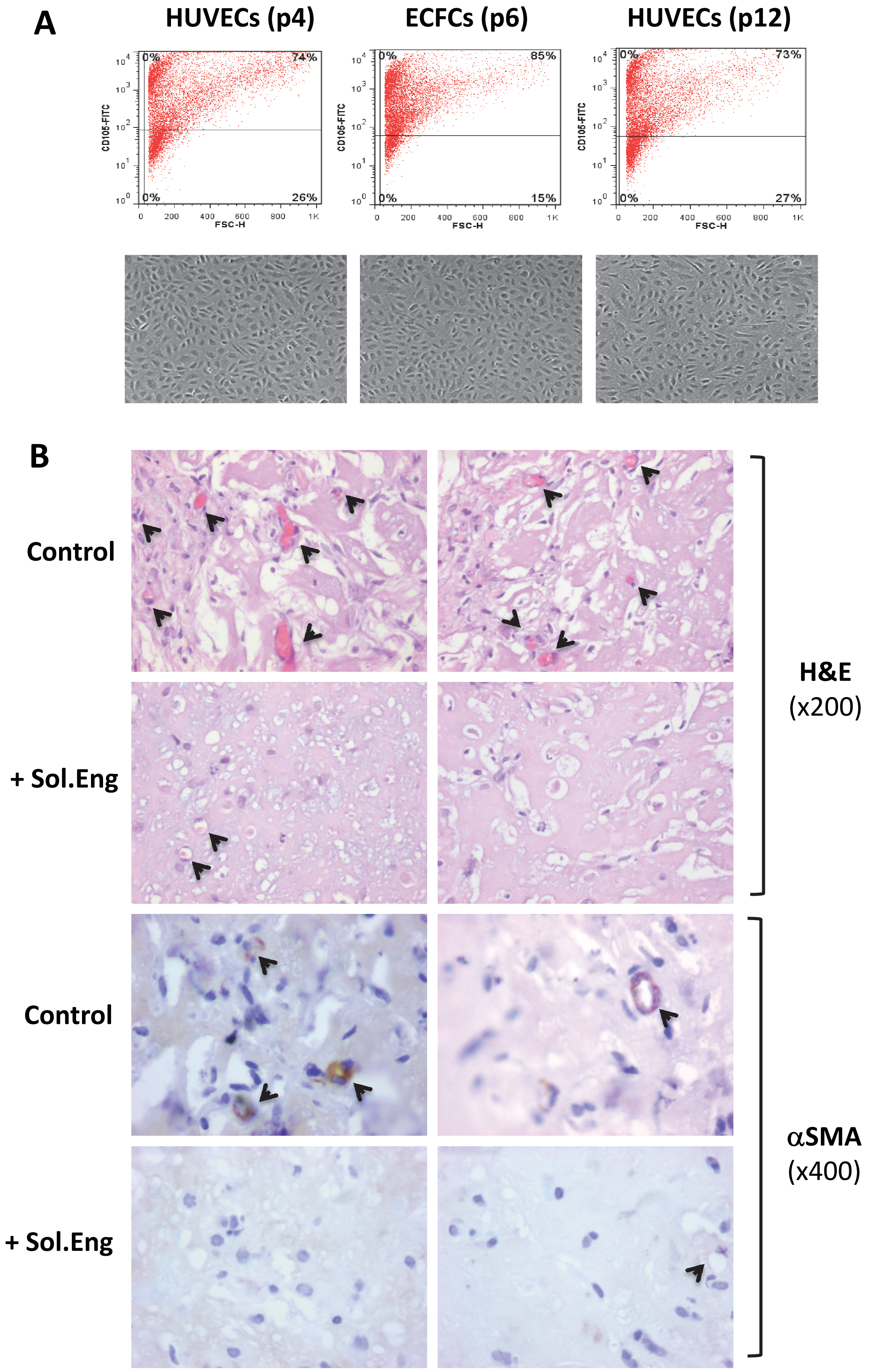

Supplemental Figure 5

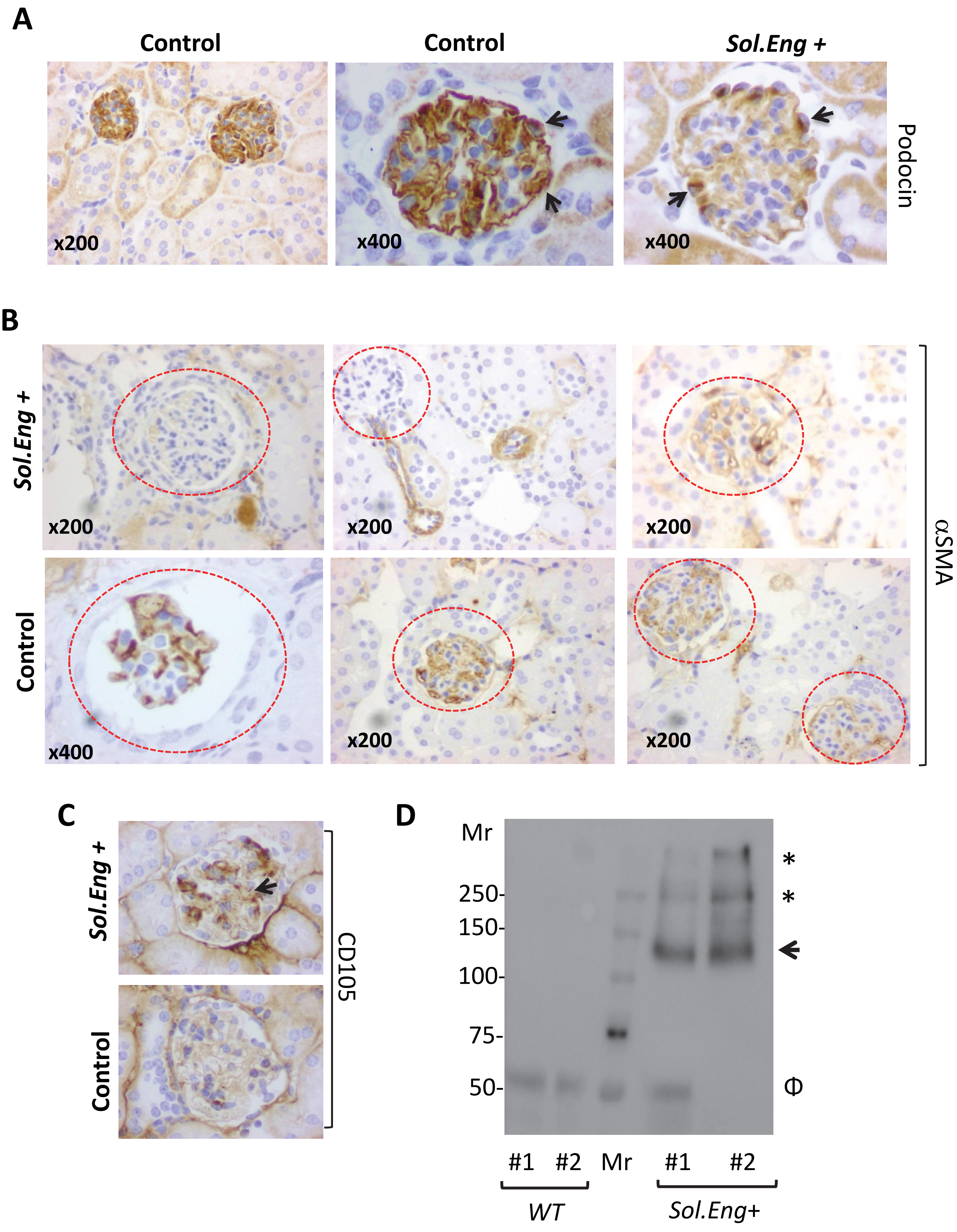

Supplemental Figure 6
